# Absolute quantitative Lipidomics reveals lipid differences in milk fat globules of yak and German Simmental cattle

**DOI:** 10.1016/j.fochx.2025.102686

**Published:** 2025-06-23

**Authors:** Yili Liu, Peng Wang, Kai Wang, Yueyue Li, Zifeng Ma, Jian Li, Daoliang Lan, Liqiang Han, Wei Fu

**Affiliations:** a Key Laboratory of Qinghai-Tibetan Plateau Animal Genetic Resource Reservation and Utilization, Ministry of Education, College of Animal & Veterinary Sciences, Southwest Minzu University, Chengdu, Sichuan 610041, China; b Sichuan Ganzi Tibetan Autonomous Prefecture, Institute of Animal Husbandry Science, Kangding 626000, Sichuan, China; c Sichuan Academy of Animal Husbandry Science, Chengdu, Sichuan 500643, China; d Key Laboratory of Animal Biochemistry and Nutrition, Ministry of Agriculture and Rural Affairs, College of Veterinary Medicine, Henan Agricultural University, Zhengzhou 450046, China

**Keywords:** Yak, Milk fat globules, Absolute quantitative lipidomics, Lipid biomarkers, Metabolic pathways

## Abstract

Lipids in milk fat globules (MFGs) are critical for nutrition and health, and yak milk, rich in milk fat, is vital for herders. This study used absolute quantitative lipidomics to compare the differences in MFG lipids in mature milk between yak (Y) and German Simmental cattle (GS). Compared with GS, Y showed higher milk fat (5.37 ± 1.70 vs. 2.31 ± 1.27 %), larger MFG size (*D*_[4,3]_: 8.69 vs. 4.31 μm). 2414 lipid species from 43 lipid subclasses in Y and GS were identified, and 187 significantly different lipids (SDLs; variable importance for the projection (VIP) > 1, fold change (FC) > 1.5 or < 0.67 with *P* < 0.05) were then screened out. TG(18:0_18:1_20:1), TG(18:1_18:3_18:3), Hex1Cer(d14:2_16:0), and TG(4:0_16:0_23:0) were potential lipid biomarkers. These SDLs involved 23 KEGG metabolic pathways, with sphingolipid metabolism being the most relevant. Our findings enhance understanding of MFG lipid differences between Y and GS, promote the development of functional yak milk product, and lay a quality identification foundation.

## Highlights

• 2414 lipid species from 43 subclasses were identified in milk fat globules of yak and German Simmental cattle milk.
• 187 significantly different lipids were screened between yak and German Simmental cattle milk.
• Four lipids were identified as potential biomarkers distinguishing yak and German Simmental cattle milk.
• Sphingolipid metabolism was the key lipid metabolic pathway.

## 1 Introduction

The yak (*Bos grunniens*), a sizable mammal, usually lives in the high-cold area at an altitude of 3000–5000 m. Currently, approximately 17.5 million yaks are widely spread across 10 countries including China, Russia, Mongolia, India, Nepal, Afghanistan, and Pakistan (Jing et al., 2022; Li, Liu, et al., 2023), Over 94 % of yaks are chiefly scattered throughout China's Qinghai-Tibet Plateau and its surrounding areas, including Qinghai, Tibetan, Sichuan, Gansu, Yunnan and Xinjiang provinces (Li, Liu et al., 2023). Yak milk, known as “natural concentrated milk”, is consumed directly or processed into high-nutrition traditional foods such as butter, cheese, ghee, yogurt and Tibetan tea (Kalwar et al., 2023; Li Zong et al., 2023). Surprisingly, high-altitude locals rarely suffer from altitude-related diseases like edema, premature aging, and atherosclerosis, possibly linked to bioactive substances such as lipids in yak milk and its derivatives (Agyare & Liang, 2021; Kalwar et al., 2023; Singh et al., 2023). Compared with ordinary milk, yak milk has higher lipid content, with its MFGs featuring larger average diameter, wider membrane surface area, and more numerous, broader-sized irregular lipid domains, highlighting distinct lipid composition and functional characteristics in yak MFGs (Agyare & Liang, 2021; Luo et al., 2018; Ma et al., 2017). Thus, comprehensive and in-depth exploration of yak MFGs' lipid profile is essential to identify potential biological functions of yak milk lipids and promote development of related functional products.

MFGs, which exist in milk as tiny globules, constitute a complex assemblage comprising proteins, cholesterol, glycoproteins, glycolipids, neutral lipids, and polar lipids (Chen et al., 2023; Maheshwari et al., 2024)‌. While MFGs from different species share structural similarities, they differ significantly in size, composition, and profile. MFG size, critical for milk stability, functionality, and nutrition, varies widely (0.1–18 μm) across species, with yak milk's average MFG size notably larger than cow milk's (Luo et al., 2016; Thum et al., 2021). Milk with larger MFGs and higher fat content coagulates more easily during processing, and yak milk's tendency to fat coalescence and creaming makes it ideal for producing butter and ghee (Ma et al., 2017; Singh et al., 2024). Moreover, most milk nutrients are carried in MFGs, which consist of a triacylglycerol (TAG) core surrounded by a three-layer milk fat globule membrane (MFGM) (Maheshwari et al., 2024). MFGMs contain bioactive molecules like cerebrosides, gangliosides, and mucins, which exhibit antibacterial, anti-inflammatory, and anticancer properties (Brink & Lönnerdal, 2020; Huang et al., 2022; Pan et al., 2023). Many studies have shown that MFGs may be important for newborn brain and neural development, digestion and absorption, protecting the intestinal mucosa, gut health, insulin sensitivity, treating chronic inflammation, and regulating blood lipids, etc., but actually, lipids are irreplaceable in these processes (Breij et al., 2019; Lee et al., 2018; Raza et al., 2021; Wang et al., 2024).

Lipids, a class of hydrophobic or amphoteric small molecules, are involved in various biological processes, such as composition of biological membrane structure (Harayama & Riezman, 2018), energy storage (Welte & Gould, 2017), cell development (Yao et al., 2020), and signal transduction (Barnett & Kagan, 2020). According to LIPID MAPS classification, lipids are categorized into 8 types: sphingolipids (SPs), glycerophospholipids (GPs), saccharolipids (SLs), sterol lipids (STs), polyketides (PKs), glycerolipids (GLs), fatty acyls (FAs), and prenol lipids (PRs), and each type is further subdivided into main classes, subclasses, and species (Fahy et al., 2009; Liebisch et al., 2020). Lipids are one of the major components of milk, scattered in the form of MFGs in milk (Macgibbon & Taylor, 2020). The major lipid categories in milk are GPs, GLs, and SPs (Li et al., 2024), followed by FAs and SLs. To date, 4000 types of lipid species have been recognized and identified in bovine milk (Liu & Rochfort, 2023). GLs such as TAGs comprise the core of these MFGs, while polar lipids (PLs) such as PE, PS, PC and PI, CH, SM, and gangliosides constitute the essential components of the MFGM (Liu et al., 2020; Maheshwari et al., 2024; Pan et al., 2022). Milk TAGs, the main component of milk fat, are vital for infants' growth, serving as an energy source, nutrient carrier, and critical for cognitive and immune development (Hanuš et al., 2018; Karrar et al., 2023). Notably, PLs, although a minor component of milk fat, are also have important functions for human health. PE, functioning as a lipid chaperone, not only facilitates the folding of specific membrane proteins but is also requisite for the activity of multiple respiratory complexes and assumes a critical role in the initiation of autophagy (Patel & Witt, 2017). Interactions of CH, SM, and SP form lipid rafts involved in membrane transport, cell signaling, lipid and protein sorting, and brain development (Hussain et al., 2019; Jiang and Li, 2022). In addition, research has found that, apart from physical and chemical identification methods (Gao et al., 2022), SDLs are also potential lipid biomarkers for identifying the authenticity of dairy products (Wang, Liu, et al., 2023; Wu et al., 2023). However, it should be emphasized that the complexity of the milk fat system, stemming from the diversity of fatty acid species and their positional distribution, has long impeded the systematic exploration of the correlation among its composition, structure, and function.

Lipidomics, a branch of metabolomics, uses high-throughput analysis techniques to systematically analyze the differences in lipid composition and expression in living organisms and efficiently study lipid family/molecule changes and functions in various biological processes (Avela & Siren, 2020). Liquid chromatography-mass spectrometry (LC-MS) stands as the preeminent technique for milk lipidomics analysis (Liu et al., 2018). In recent years, lipidomics has been extensively applied to identify molecular species, elucidate deep structures, and accurately quantify milk lipids (Liu & Rochfort, 2023). Notably, considerable attention has been devoted to potential factors influencing milk fat composition and function, including different species (Wang, Zhou, et al., 2023; Wu et al., 2023), geographical regions (Shang et al., 2022), lactation periods (Li, Li, Kang, et al., 2020; Li et al., 2024), diets (Deng et al., 2022), processing methods (Zhang et al., 2022). Based on liquid chromatography-tandem mass spectrometry (LC-MS/MS), the differences of lipid nutrients in milk of 13 species are analyzed, and the results show that the content of short-chain fatty acids in ruminants was higher than that in non-ruminants and PS (22:5_18:2) was identified as a potential biomarker in porcine milk (Wu et al., 2023). Some PLs with significant differences are also identified as biomarkers to distinguish breast milk from ewe milk by UHPLC-QTRAP-MS-based lipidomic analysis (Wang et al., 2023). Based on UHPLC-Qtrap-MS, 41 SDLs are identified in yak colostrum and mature milk, among which four potential lipid biomarkers were identified (Li et al., 2024). Although a few studies have described the lipid composition of yak milk and its milk products (Li et al., 2024; Yang et al., 2024), mainly for whole milk, the lipid profiles of MFGs have not been thoroughly evaluated for yak and German Simmental cattle mature milk. We hypothesized that lipidomics could identify lipid disparities in MFGs between yak and German Simmental cattle milk, and screen for potential distinguishing biomarkers.

Therefore, the present study aimed to conduct a comprehensive identification and quantification of lipids in the mature milk of yak and German Simmental cattle through the application of absolute quantitative lipidomics relying on the UPLC-Orbitrap mass spectrometry system, as well as to screen for and validate SDLs and potential lipid biomarkers for the purpose of differentiating between the two. These results can offer crucial support for evaluating the nutritional value of yak and German Simmental cattle milk. These results will also fill the gaps in the lipid profiles of their MFGs, providing comprehensive lipid data for authenticating yak milk's nutritional quality and developing functional yak MFG products. Furthermore, they will lay a theoretical foundation for creating an accurate, rapid, and cost-effective method to verify the quality and authenticity of yak milk products, with significant practical implications.

## 2 Material and methods

### 2.1 Instruments and reagents

The product information used was listed below: Q-Exactive Plus Mass Spectrometer (Thermo Scientific); UHPLC Nexera LC-30 A Ultra High Performance Liquid Chromatograph (SHIMADZU); Low temperature high speed centrifuge (Eppendorf 5430R); The columns were Waters, ACQUITY UPLC CSH C18, 1.7 μm, 2.1 mm × 100 mm column; acetonitrile, isopropyl alcohol and methanol (Thermo Fisher); Methyl tert-butyl ether (MTBE,SIGMA); Internal Standards for 14 isotopes: SPLASH® LIPIDOMIX MASS SPRC STANDARD, AVANTI, 330707-1EA.

### 2.2 Experimental design and sample collection

**Fig. 1:**
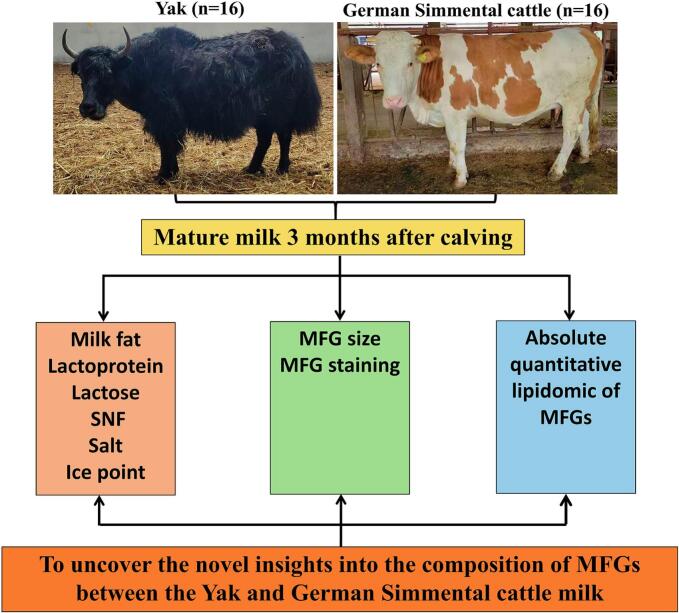
Schematic diagram of experimental design.

All experimental procedures involving animals were reviewed and approved by the Academic Morality and Ethical Committee at Southwest Minzu University (Chengdu, Sichuan, China) (permit number: smu-202401164), and all studies were in line with the requirements of the directory of the Ethical Treatment of Experimental Animals of China. 32 milk samples (3 months postpartum) were collected from 16 Changtai yaks and 16 German Simmental cattle. Healthy female yaks (3–6 years old, 280–320 kg) in Baiyu County, Garze Tibetan Autonomous Prefecture, Sichuan Province, were naturally grazed without supplementary feeding. Healthy female German Simmental cattle (3–6-year-old, 460–520 kg) in Meishan City, Sichuan Province, were raised under normal house feeding. Following manual milk extraction, the milk was filtered through disposable gauze into sterile freezi ng tubes. Subsequently, it was promptly transported to the laboratory in an ice-box and stored at −80 °C until analysis. Each sample was stored individually without mixing. The experimental design is presented in Fig. 1.

### 2.3 Determination of milk composition

Milk fat, lactoprotein, lactose, solid not fat (SNF), salt and freezing point were measured in 32 milk samples by using Ultrasonic Milk Analyzer (Master LM2, Milkotester Ltd., Bulgaria). The measurement process is carried out according to the instrument manual.

### 2.4 Measurement of MFG size

A laser particle size analyzer was employed to ascertain the dimensions of MFGs in fresh milk (Xing et al., 2020). Specifically, the wet measurement mode of the Mastersizer 3000 laser particle size analyzer was utilized, with the laser refractive index being fixed. 1 mL of milk was added to 400 mL of water, mixed thoroughly, and then injected into the instrument. The system automatically generated the average MFG size derived from three independent measurements. The system automatically generated average data from three repeated measurements, including volume-equivalent diameter (*D*_[4,__3]_), surface-area-equivalent diameter (*D*_[3,2]_), and specific surface area (SSA).

### 2.5 Staining of MFG

1 mL of fresh milk was diluted with an equal volume of water. Subsequently, 200 μL of a 1 % Nile red solution and 4 μL of a 0.1 % fluorescein isothiocyanate solution were introduced. After thorough homogenization, the mixture was gently agitated in the absence of light for 20 min. Thereafter, it was combined with agarose and transferred onto a microscopy slide to observe the localization of triglycerides in the MFG core and membrane proteins.

### 2.6 Lipid extraction

The MFGs were extracted from equal-volume yak milk and German Simmental cattle milk respectively by using the operating instructions of APTBIO (Shanghai, China). During the test, every 3 samples were mixed into 1 pool, and 16 samples were mixed into 6 pools (with 2 samples mixed twice). The main steps were as follows: 1) An appropriate amount of milk of equal volume was taken into each centrifuge tube, and centrifuged at 10,000 rcf for 15 min to obtain the upper layer sample; 2) 10 mL PBS was added for three washes, and ultrasonic treatment was conducted once (80 w, 15 s) between each wash; 3) Centrifugation was performed again at 10,000 rcf for 1 h at 4 °C, and the upper layer MFG sample was collected into a new centrifuge tube for later use. Subsequently, the extraction of lipids from the MFGs was carried out as described below: 1) An appropriate volume of the above sample was taken, 200 μL of water and 20 μL of the internal lipid standard mixture were added, and the mixture was vortexed; 800 μL of MTBE was introduced, and the mixture was vortexed again to ensure thorough mixing；2) 240 μL of pre-cooled methanol was added, and the solution was gently swirled. The mixture was placed in a low-temperature water bath for ultrasonic treatment for 20 min, then allowed to stand at room temperature for 30 min. The sample was centrifuged at 10 °C for 15 min, and the upper organic layer was carefully collected and dried under a stream of nitrogen；3) For mass spectrometry analysis, the dried residue was reconstituted in 200 μL of 90 % isopropyl alcohol/acetonitrile and vortexed thoroughly. 90 μL of the solution was taken, centrifuged at 14,000 *g* and 10 °C for 15 min, and the supernatant was collected for analysis.

### 2.7 Lipid analysis

Samples were separated using a UHPLC Nexera LC-30 A ultra-high performance liquid chromatography system. A C18 chromatographic column was used, with a column temperature of 45 °C and a flow rate of 300 μL/min. The mobile phases consisted of two components. Mobile phase A was an acetonitrile-water mixture (acetonitrile:water = 6:4, *v*/v), supplemented with 0.1 % formic acid and 0.1 mM ammonium formate. Mobile phase B was an acetonitrile-isopropyl alcohol mixture (acetonitrile:isopropyl alcohol = 1:9, v/v), also containing 0.1 % formic acid and 0.1 mM ammonium formate. The gradient elution program was set as follows: from 0 to 3.5 min, the proportion of B was maintained at 40 %; from 3.5 to 13 min, B linearly changed from 40 % to 75 %; from 13 to 19 min, B linearly changed from 75 % to 99 %; from 19 to 24 min, B was maintained at 40 %. Throughout the analysis, the samples were placed in an automatic injector maintained at 10 °C. To counteract any potential interference from fluctuations in the instrument's detection signal, these samples were analyzed continuously in random order.

Detection was performed using electrospray ionization (ESI) in both positive and negative ion modes. Samples were first separated by UHPLC and then analyzed by a QExactive series mass spectrometer. ESI source conditions were as follows: Heater Temp 300 μL, Sheath Gas Flow rate 45 arb, Aux Gas Flow Rate15 arb, Sweep Gas Flow Rate 1arb, spray voltage 3.0 KV, Capillary Temp 350 °CS-Lens RF Level 50 %, MS1 scan ranges: 200–1800. The mass charge ratio of lipid molecules and lipid fragments was collected as follows: 10 fragment maps (MS2scan, HCD) were collected after each full scan. At *M*/*Z* 200, the resolutions of MS1 and MS2 were 70,000 and 17,500 respectively.

### 2.8 Lipid data processing

LipidSearch is a search engine for lipid species identification relying on MS/MS matching. The LipidSearch database encompasses over 30 lipid classes and 1.5 million fragment ions. Both precursor and fragment mass tolerances were set at 5 ppm. LipidSearch was used for peak identification, extraction, and lipid identification (secondary identification). Key parameters included: precursor tolerance of 5 ppm, product tolerance of 5 ppm, and product ion threshold of 5 %. First, the quality of the data extracted by LipidSearch was evaluated, and only the qualified data was further analyzed. Data analysis included identification quantity statistics, lipid composition analysis, and lipid difference analysis. Lipid composition analysis covered lipid subclass composition and lipid content distribution. Lipid difference analysis involved lipid content, chain length, and chain saturation. The analysis of lipid content changes was conducted across multiple dimensions, including the whole lipidome, subclasses, and molecules.

### 2.9 Statistical analysis

GraphPad Prism 8 was used for analyzing milk composition and MFG size. *t*-tests were applied for pairwise group comparisons. Following univariate analysis, differential expression analysis was performed on all detected lipid molecules. Results were visualized in a volcano plot, where lipid molecules with a FC > 1.5 or FC < 0.67 and *P* value <0.05 were color-coded. Multidimensional statistical analyses, including principal component analysis (PCA), partial least squares discrimination analysis (PLS—DA), and orthogonal partial least-squares discrimination analysis (OPLS-DA), were conducted on the dataset. The VIP values obtained from the OPLS-DA model were used to evaluate the impact of lipid molecule expression patterns on sample group classification and interpretation and to identify biologically significant differential lipid molecules. Lipids with VIP > 1.0 were considered to make significant contributions to model interpretation. Thus, VIP > 1.0, FC < 0.67 or FC > 1.5, and *P* value <0.05 were established as the screening criteria for SDLs. These SDLs were used to screen for potential lipid biomarkers. The lipid-lipid correlation matrix of SDLs was converted into a chord diagram, depicting lipid molecular pairs with a correlation coefficient |r| > 0.8 and *P* value <0.05 (Aviram et al., 2016). Correlation network construction and KEGG pathway enrichment analysis of SDLs were performed using Cytoscape 3.7.1 (Seattle, WA, USA) and the Kyoto Encyclopedia of Genes and Genomes (https://www.kegg.jp/kegg/), respectively. MetaboAnalyst 6.0 (http://www.metaboanalyst.ca) was utilized for lipid metabolomic visualization.

## 3 Results and discussion

### 3.1 Milk composition

**Fig. 2:**
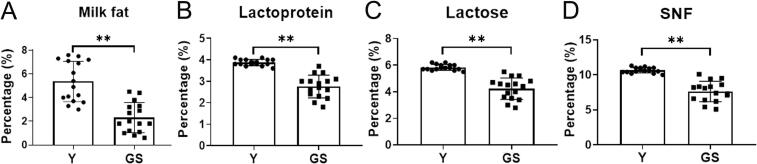
Determination of milk fat (A), lactoprotein (B), lactose (C) and SNF (D) in Y and GS. Y = Yak milk; GS = German Simmental cattle milk. *n* = 16. ** means *P* < 0.01.

Yak milk and German Simmental cattle milk were analyzed for milk composition content, including milk fat, lactoprotein, lactose, SNF, salt, and freezing point. The milk fat, lactoprotein, lactose, SNF, salt and freezing point in Y were significantly higher than those in GS (*P* < 0.01; Fig. 2 and Fig. S1). In particular, the milk fat content of Y (5.37 ± 1.70 %) was more than 2-fold that of GS (2.31 ± 1.27 %) (Fig. 2A). Milk fat, an important constituent of milk, plays a crucial role by providing energy and essential fatty acids, determining the flavor of milk, and directly influencing the nutritional, physical, and sensory properties of milk and dairy products (Waldron et al., 2020; Wolf et al., 2013). The high fat content, recognized as one of the most remarkable features, is crucial for distinguishing yak milk from other milks(Wang et al., 2023). Numerous studies have shown that yak milk has a significantly higher average fat content than cow, goat, horse, and human milk (Li et al., 2023; Wen et al., 2017).

### 3.2 MFG size and staining

**Fig. 3:**
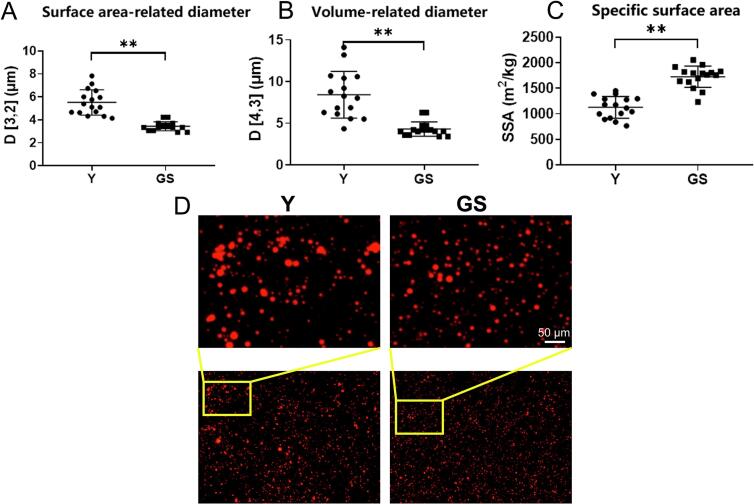
MFG size *D*_[3, 2]_ (A), *D*_[4,3]_ (B) and SSA (C) and staining of MFGs (D) in Y and GS. Y = Yak milk; GS = German Simmental cattle milk. *n* = 16. ** means *P* < 0.01.

Milk fat exists in the form of fat globules with varying particle sizes.Compared with GS, the mean values of *D*_[3, 2]_ (5.52 μm vs. 3.44 μm) and *D*_[4, 3]_ (8.69 μm vs. 4.31 μm) were higher in Y (*P* < 0.01; Fig. 3A and B), but the SSA values (1127.13 m^2^kg^−1^ vs. 1726.25 m^2^kg^−1^) were significantly lower (*P* < 0.01; Fig. 3C). The structure of the MFGs was depicted in Fig. 3D. Compared with GS, the proportion of large milk fat globules in Y was higher, and the proportion of small milk fat globules was lower (Fig. 3D). In a comparison, equine milk is found to have the smallest fat globules, whereas buffalo milk boasts the largest (Gantner et al., 2015). The size of yak MFG is larger than that of mare, donkey, human, camel, reindeer, cow, goats, ewe, and sheep, but smaller than that of buffalo (Luo et al., 2018; Moatsou, 2019). However, due to the high fat content and large-sized MFGs in yak milk, these globules are rendered unstable and prone to rupture under mechanical stress. This rupture can, in turn, promote a greater degree of coalescence of milk fat during the processing, thereby streamlining the cheese-making process (Luo et al., 2018). Yak milk, characterized by a high concentration of fat globules and a relatively large average diameter, stands out as an optimal raw material for the processing of butter and ghee (Li et al., 2023). Previous studies reveal that yak MFG exhibits superior lipolysis ability and higher digestion efficiency than cow MFG in in vitro infant gastrointestinal digestion, primarily due to its lipid properties that significantly offset the drawbacks of its larger size (Luo et al., 2020).

### 3.3 Validation of analytical methods

This experiment thoroughly assessed the stability of the instrument, the reproducibility of the experiment, and the dependability of data quality via six quality control aspects. These aspects consisted of comparing the Base Peak spectrum of QC samples (BPC), analyzing the correlation spectrum of QC samples, performing principal component analysis on the total sample (PCA), carrying out the Hotelling's T2 test on the total sample, multivariate control chart of QC sample, and relative standard deviation (RSD) of QC samples. The lipid extracts from 6 Y samples and 6 GS samples were equally divided and mixed to produce 3 QC samples. Comparison of the base peak chromatograms and retention times of three QC samples in positive and negative ion modes revealed good overlap, suggesting stable detection performance of the mass spectrometry system and excellent experimental repeatability (Fig. S2). The results of Pearson correlation analysis performed on QC samples indicated that all the correlation coefficients among these samples were above 0.9 (Fig. S3A). The ion peaks obtained from all experimental and QC samples were subjected to Pareto-scaling and subsequently PCA analysis, and the outcomes demonstrated that QC samples were tightly grouped (Fig. S3B and 3C). Hotelling's T^2^ test was carried out on all samples (Fig. S3D), and the findings indicated that every QC sample fell within the 99 % confidence interval (Siskos et al., 2017). The above results demonstrated the excellent repeatability of the experiment. The multivariate control chart (MCC) of the QC samples revealed that the fluctuations of these samples stayed within the range of plus or minus three standard deviations. This finding indicated that the instrument's fluctuations were within the normal range (Fig. S3E). Moreover, the peaks with a relative standard deviation (RSD) of 30 % or less made up over 80 % of the total peaks in the QC samples (Fig. S3F). The above results suggested that the instrument analysis system had high-level stability, which meant the data were suitable for further analysis (Shen et al., 2016).

### 3.4 Lipid composition analysis of Y and GS

**Fig. 4:**
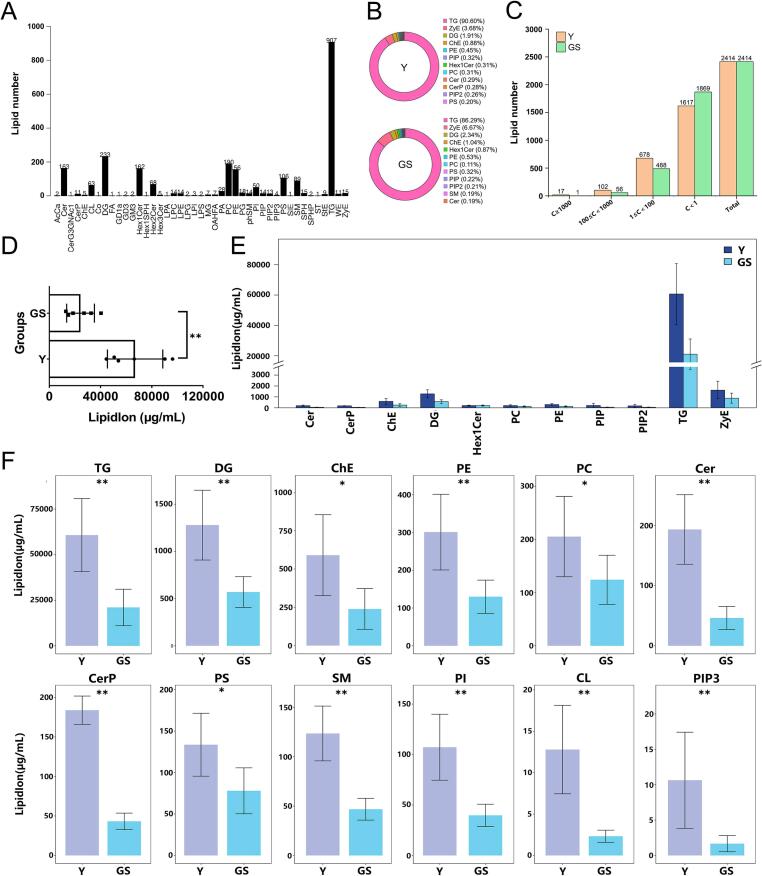
The number and content of lipids species identified in lipid subclasses. Number of lipid species identified in 43 lipid subclasses (A). The pie chart of the main lipids classes in Y and GS (B) (Complete data are presented in Fig.S4, and the same pie chart is used in those two figures). The number of lipid species in different concentrations in Y and GS (C). The content of total lipids between Y and GS (D). Comparison of high concentration lipid subclasses between Y and GS (E). Comparison of lipid subclasses with significant differences between Y and GS (F). Y = Yak milk; GS = German Simmental cattle milk. *n* = 6. * means *P* < 0.05, ** means *P* < 0.01.

A sum of 2414 lipid species was recognized from Y and GS, and they were divided into 43 lipid classes, including 907 TGs, 233 DGs, 190 PCs, 163 Cers, 162 Hex1Cers, 106 PSs, 89 SMs, 68 Hex2Cers, 63 CLs, 56 PEs, 50 PIs, 28 PAs, 18 PGs, 15 ZyEs, 15 SPHs, 14 LPCs, 14 LPEs, 14 PIPs, 14 phSMs, 13 PIP2, 11 WEs, and 11 CerPs, etc. (Fig. 4A). Notably, TGs, DGs, PCs, Cers, Hex1Cers, PSs, and SMs were the most dominant lipid classes. This finding is comparable to the composition of major lipids in yak colostrum and mature milk but significantly different from that in human, ewe, and goat colostrum (Li et al., 2024; Wu et al., 2023; Xiong et al., 2023; Xiong et al., 2024). However, FFA was not detected in this study because it was based on the absolute quantitative analysis of the extracted MFGs. FFA is part of the milk fat composition, but not in the form of milk fat globules (Hurtaud et al., 2020; Wiking et al., 2006). This study found that the main lipids classes in Y included 90.60 % TGs, 3.68 % ZyEs, 1.91 % DGs, 0.88 % ChEs, 0.45 % PEs, 0.32 % PIPs, 0.31 % Hex1Cers, 0.31 % PC, 0.29 % Cer, 0.28 % CerPs, 0.26 % PIP2s, and 0.20 % PSs (Fig. 4B), and those in GS included 86.29 % TGs, 6.67 % ZyEs, 2.34 % DGs, 1.01 % ChEs, 0.87 % Hex1Cers, 0.53 % PEs, 0.32 % PSs, 0.22 % PIPs, 0.21 % PIP2s, 0.19 % SMs, 0.19 % Cers, and 0.11 % PCs (Fig. 4B). In addition, there were 31 other lipid classes with lower proportions (Fig. S4). TGs represent the most predominant lipid component in milk, and their proportion is higher than 90 % in almost all milks, such as human milk, cow milk, goat milk, sheep milk, and camel milk, etc. (Zhao et al., 2022). However, the distribution of other lipid subclasses varies significantly depending on milk source and lactation period (Hewelt-Belka et al., 2020; Wang et al., 2023).

Furthermore, significant differences existed in the concentrations of different lipid species between the two types of milk (Fig. 4C). In Y and GS, the total number of lipid species with concentrations greater than 1 μg/mL were 797 and 545, respectively, among which 17 and 1 species were greater than 1000 μg/mL, 102 and 56 were between 100 and 1000 μg/mL, and 678 and 488 were between 1 and 100 μg/mL. The dynamic distribution of lipid content was analyzed, and TG(16:0_6:0_18:1) and TG(10:0_10:0_14:0) were the most abundant in Y and GS, respectively, while CL(61:11) had the lowest concentration in both Y and GS (Fig. S5). The top 20 lipid molecules were analyzed in Y and GS, and the results showed that except for ZyE(33:6) and ZyE(23:6), the abundance of other lipid molecules in Y was significantly higher than that in GS (*P* < 0.05) (Table S1). Among those, TG(16:0_6:0_18:1), TG(4:0_18:1_18:1), TG(16:0_6:0_16:0), TG(16:0_10:0_14:3), TG(16:0_10:0_14:3), TG(8:0_10:0_12:0), TG(4:0_16:0_18:2), and TG(16:0_6:0_14:0) in Y were more than three times those in GS (Table S1). These findings may be closely related to the unique flavor of yak milk (Xiong et al., 2024). KEGG enrichment analysis of all lipid molecules in Y and GS revealed that the top 20 signal pathways were identical and showed significant enrichment (*P* < 0.05) (Fig. S6 and Table S2). Notably, three of these pathways were linked to lipid metabolism, namely glycerophospholipid metabolism, sphingolipid metabolism, and glycerolipid metabolism (Fig. S6 and Table S2).

### 3.5 Difference analysis of lipid content between Y and GS

As shown in Fig. 4D, the total lipid content in Y was significantly higher than that in GS (*P* < 0.01). As shown in Fig. 4E, F, S7, and S8, the content of different lipid subclasses of Y and GS was compared, among which 25 showed significant differences. The total content of TGs, DGs, PEs, Cers, CerPs, ChEs, PCs, PSs, Hex1SPHs, LPAs, LPCs, LPEs, SPHs, WEs, SMs, PIs, CLs, PIP3s, Cos, LPIs, MGs, OAHFAs, and phSMs in Y was significantly higher than that in GS (*P* < 0.05) (Fig. 4F, S8, and Table S3), whereas the total content of GD3s and Hex3Cers in Y was lower than that in GS (*P* < 0.05) (Fig. S8 and Table S3), and the content of other lipid molecules showed no significant difference between Y and GS (Table S3). These results are similar to those obtained in yak colostrum and mature milk (Li et al., 2024). That study also found that these lipids with the top 8 lipid content included TAGs, DAGs, SMs, CEs, PEs, PSs, PCs, and PIs in yak colostrum and mature milk (Li et al., 2024). However, the corresponding concentrations in this study were significantly higher, which may be attributed to differences in methodology. In addition, NLs such as TGs and CEs constitute the core of MFGs, while PLs such as GPs and SMs wrap MFGs and constitute the basic components of MFGM, which can further explain why the proportion of larger MFGs in Y was higher in this study. Moreover, it was also found that the concentrations of polar lipids (PLs) (such as PE, PC, PS, SM, LPC, and PI) were significantly higher in Y. During the early growth stage, yak calves have a greater demand for polar lipids, likely because PLs play vital roles in key physiological processes, such as forming biofilms, promoting neurodevelopment, regulating lipid metabolism, maintaining intestinal ecology, influencing inflammation, and affecting cardiovascular health (Anto et al., 2020; Milard et al., 2019; Zhou & Ward, 2019). For example, SM is involved in constructing lipid rafts and the nerve myelin structure in the brain (Yuan et al., 2024; Zheng et al., 2019), and PE and PC contribute to the suppression of cholesterol absorption and intestinal maturation (Nilsson et al., 2021; Venkat et al., 2024). Therefore, we speculate that this may be related to the harsh growing environment of calves, and the composition of yak milk enables calves better adapt to the plateau environment.

### 3.6 Screening of SDLs between Y and GS

**Fig. 5:**
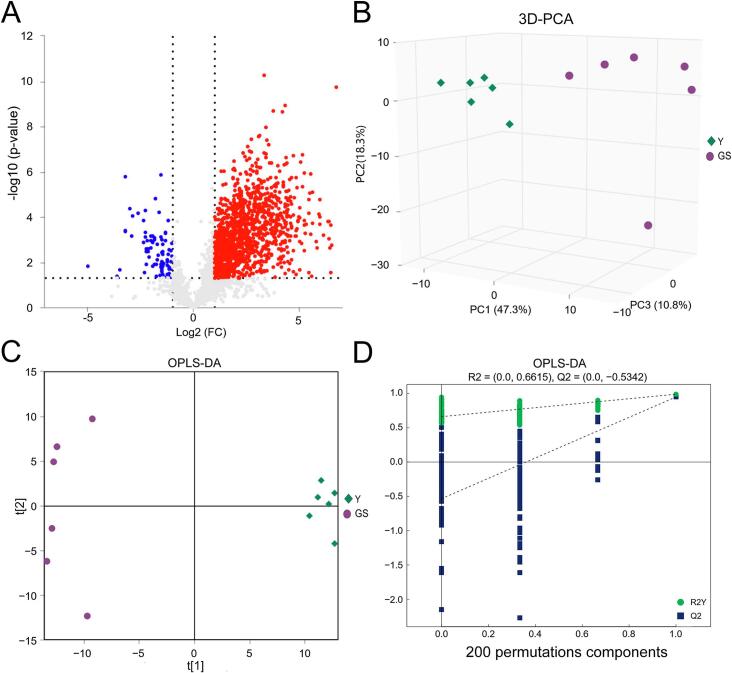
Volcano Plot (A), 3D-PCA score map (B), OPLS-DA score plot (C), and corresponding validation plots of OPLS-DA (D). Y = Yak milk; GS = German Simmental cattle milk; t[1] represents principal component 1, and t[2] represents principle component 2; R2Y represents the interpretation rate of the model to the Y variable; Q2 indicates the predictive power of the model. The abscissa stands for the permutation retention, with the ordinate indicating the values of R2 and Q2 in D.

To further explore the overall distribution of milk lipids and to screen for differential lipids in Y and GS, we performed a multi-level statistical analysis on the milk lipid identification data, including univariate statistical analysis (FC Analysis and *t*-test/non-parametric test) and multivariate statistical analysis (PCA, PLS-DA, and OPLS-DA). Using univariate statistical analysis, all detected lipid molecules were analyzed for differences. The screening criteria were set as lipid molecules with an FC > 1.5 or < 0.67 and a *P* value <0.05. The analysis results are presented in a volcano plot, showing that most lipid molecules were upregulated in Y (Fig. 5A). In total, 1116 differential lipid molecules were identified, including 376 TGs, 133 DGs, 86 Cers, 81 PEs, 69 PCs, 68 Hex1Cers, 51 SMs, 45 CLs, 43 PSs, 36 Hex2Cers, 35 PIs, and others (Table S4). The score plots of 3D-PCA and OPLS-DA for lipids in Y and GS were presented in Fig. 5B and C, respectively. Based on the 3D-PCA score plot, Y and GS exhibited clear separation (Fig. 5B), indicating the reliability of the analysis. To achieve a greater degree of group separation and gain a more comprehensive understanding of the variables contributing to classification, supervised OPLS-DA was applied (Fig. 5C). A 7-fold cross-validation was conducted to assess the robustness and predictive capacity of our model, from which the model evaluation parameters R^2^Y and Q^2^ were derived. In the OPLS-DA model, the values of R^2^Y and Q^2^ were 0.988 and 0.948 respectively. A permutation test was implemented to verify the model and ensure the validity of the OPLS-DA model. In the OPLS-DA permutation test, the R^2^ and Q^2^ values were (0.0, 0.6615) and (0.0, −0.5342), respectively (Fig. 5D). These findings suggested that as the replacement retention decreased, the R^2^ and Q^2^ of the random model gradually declined, demonstrating that both the PLS-DA and OPLS-DA models were robust and free from over-fitting.

**Fig. 6:**
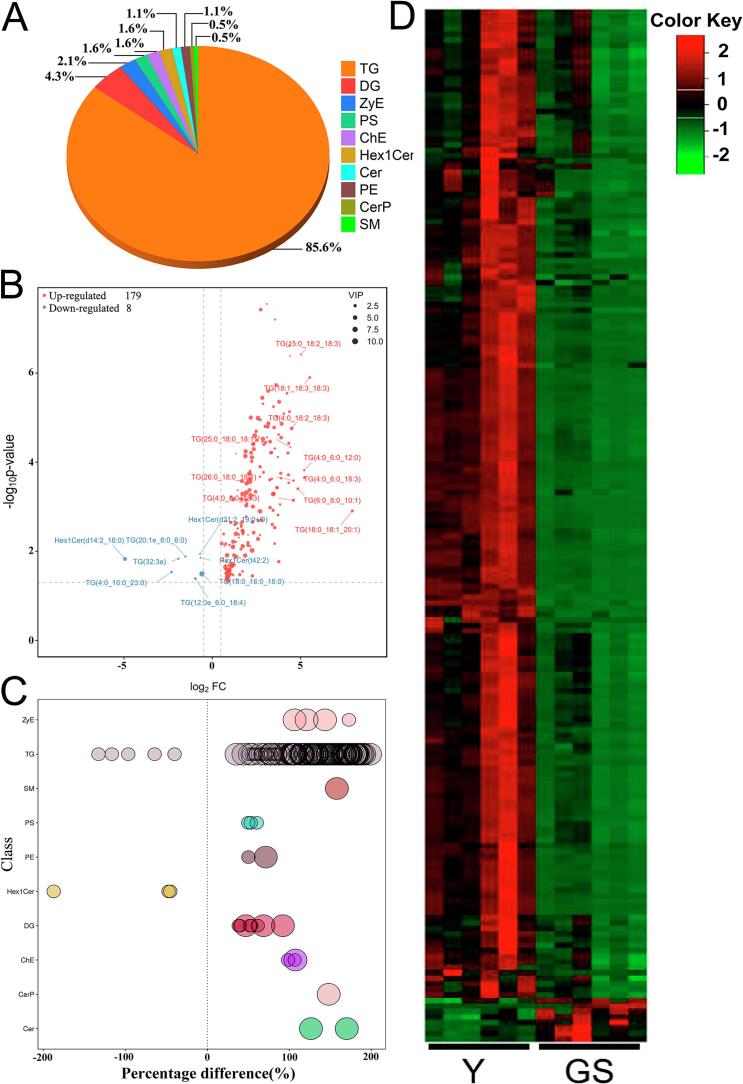
Percentages of numbers of SDLs subclasses between Y and GS (A), the percentages (%) in the pie-chart mean the number of each lipid subclass to the total SDLs. Volcano plot of lipids of SDLs subclasses between Y and GS (B). The bubble diagram for SDLs in Y and GS (C). The bubbles represent the SDLs; the ordinate represents the lipid subclass; the bubble size represents the difference significance of SDLs; these smaller bubbles represent the significant difference (*P* < 0.05), while these larger bubbles represent the very significant difference (*P* < 0.01). Heatmap analysis of 187 significantly different lipids between Y and GS (D).

In the present study, screening criteria of OPLS-DA VIP > 1 and a *P* value <0.05 were adopted to further screen SDLs. A total of 187 SDLs belonging to 10 lipid subclasses were identified (Table S5), including 160 TGs, 8 DGs, 4 ZyEs, 3 PSs, 3 ChEs, 3 Hex1Cers, 2 Cers, 2 PEs, 1 CerP, and 1 SM (Fig. 6A). Subsequently, a volcano plot and a bubble diagram were created to visualize the differences in SDL levels and evaluate their statistical significance (Fig. 6B and C). The results showed that the number of significantly up-regulated SDLs was much higher than that of down-regulated SDLs. Specifically, 179 SDLs were significantly up-regulated (VIP > 1, *P* < 0.05, and FC > 1.5), and 8 SDLs were significantly down-regulated (VIP > 1, *P* < 0.05, and FC < 0.67) in Y compared to GS (Table S5). Among these SDLs, several lipids exhibited significantly higher levels in Y than in GS, such as TG(18:0_18:1_20:1) (FC = 244.22), TG(18:1_18:3_18:3) (FC = 45.99), TG(4:0_6:0_18:3) (FC = 37.33), TG(4:0_6:0_12:0) (FC = 36.80), TG(15:0_18:2_18:3) (FC = 32.62), ZyE(37:6) (FC = 13.64), Cer(d18:1_23:0) (FC =12.34), SM(d16:0_20:0) (FC = 8.52), CerP(d39:1) (FC = 6.69), ZyE(35:6) (FC = 6.08), Cer (d18:1_24:0) (FC = 4.43), and ZyE(36:6) (FC = 4.06)(Table S5, *P<*0.05). However, TG(18:0_16:0_16:0) (FC = 0.67), TG(4:0_16:0_23:0)(FC = 0.20), and Hex1Cer (d14:2_16:0)(FC = 0.03) were significantly lower in Y than in GS (*P*<0.05)(Table S5). Additionally, hierarchical cluster analysis was performed to intuitively display and analyze the variation between Y and GS and the expression abundance of SDLs in different samples (Fig. 6D). The results showed clear clustering between the 6 parallel Y samples and the 6 GS samples, indicating high confidence and reproducibility in the experiment and analysis. Notably, 160 significantly different TG species were identified, with total carbon numbers ranging from 22 to 62 (Fig. S9) and double bond numbers distributed from 0 to 9 (Fig. S10). These TGs were dominated by long-chain saturated fatty acids (SFAs) (palmitic acid (PA, C16:0), stearic acid (SA, C18:0)), and followed by long-chain monounsaturated fatty acids (MUFAs) (oleic acid (OA, C18:1) and long-chain polyunsaturated fatty acids (PUFAs) (linoleic acid (LA, C18:2), linolenic acid (LNA, C‌18:3), arachidonic acid (ARA, C20:4), and eicosapentaenoic acid (EPA, C20:5)), and followed by short-chain and medium-chain SFAs (butyric (C4:0), caproic (C6:0), caprylic (C8:0), and pentadecanoic acid (PDA, C15:0)) (Table S6). This result is consistent with previous studies on yak milk fatty acids (Ding et al., 2013; Yang et al., 2022). Furthermore, compared with GS, Y contained higher levels of short−/medium−/long-chain SFAs, MUFAs, and PUFAs, which have better regulatory effects on human health. This suggests that yak milk may have more advantages in TAG composition as a substitute for breast milk (Agyare & Liang, 2021; Li et al., 2023).

The nutritional properties of milk fat are significantly determined by its fatty acid composition, which serves as a crucial factor (Hanuš et al., 2018). Previous studies have likewise indicated that yak milk contains an abundance of fatty acids, encompassing SFAs, MUFAs, and PUFAs (Agyare & Liang, 2021; Ma et al., 2017; Pal et al., 2024; Wang et al., 2023). However, lipidomics analysis of yak MFGs via extraction has not been previously reported, and this study obtained many interesting results. The content of SFAs in lipid classes such as TG, Cer, DG, Hex1SPH, Hex2Cer, LPA, LPE, LPI, MG, PE, PI, PIP3, PS, SM, and SPH was significantly higher in Y than in GS (*P*<0.05) (Fig. S11). Among 187 SDLs, 26 SFAs were up-regulated, including 24 TGs (e.g., 4:0_6:0_12:0, 6:0_8:0_8:0, 18:0_16:0_21:0), 1 SM(d16:0_20:0), and 1 PE(18:0_16:0), whereas 2 SFAs were down-regulated (TGs 18:0_16:0_16:0 and 4:0_16:0_23:0) (Table S5). This result aligns with other reports on yak milk composition (Wang et al., 2022).

For many years, SFAs (“hard fats”) have been widely demonized, leading consumers to avoid dairy products such as yak butter (Waehler, 2021). This stems from earlier studies linking high SFA intake to increased risks of metabolic syndrome and cardiovascular disease (CVD) (Cascio et al., 2012; Guasch-Ferré et al., 2015; Unger et al., 2019), with long-chain SFAs like palmitic, lauric, and stearic acids being particular health concerns (Flock & Kris-Etherton, 2013; Gupta et al., 2012; Zong et al., 2016). This narrative has driven recommendations for fat-free or low-fat dairy products (Da Silva Lannes & do Amaral, 2015; Lock et al., 2014). However, recent research has reassessed the association between SFAs and CVD risk, revealing that earlier conclusions are less definitive than once thought (Astrup et al., 2020; Givens, 2023; Jimbo et al., 2020; Ruiz-Núñez et al., 2016). Multiple studies now suggest that full-fat dairy products may offer health benefits, with some even reporting a modest negative association between full-fat dairy intake and CVD risk (Astrup et al., 2016; Bhattacharjee et al., 2020; Hirahatake et al., 2020). Notably, among SFAs, only four—palmitic, lauric, myristic, and stearic acids—have been linked to potential CVD risk in humans (Chei et al., 2018; Guasch-Ferré et al., 2015; Zhuang et al., 2019). Even so, high stearic acid intake has not been associated with increased risks of ischemic heart disease (IHD) or myocardial infarction (MI) (Praagman, Beulens, et al., 2016; Praagman, de Jonge, et al., 2016). Moreover, these four fatty acids exhibit surprising health benefits (Ameena et al., 2024; Carta et al., 2017; Khalil et al., 2021; Lappano et al., 2017). For instance, lauric acid demonstrates antimicrobial activity, drug-delivery potential, and utility in tissue-engineered scaffolds through *in vitro* and *in vivo* studies (Ameena et al., 2024). Stearic acid-rich diets have been shown to reduce fasting levels of coagulation factor VII (a blood-clotting mediator), induce apoptosis in breast cancer cells, and protect against Parkinson's disease neurodegeneration (Sabre, 2024). Myristic acid, meanwhile, safeguards testicular function against oxidative stress and inflammation in diabetic rats while lowering blood glucose and inhibiting NF-κB-mediated inflammation in microglia (Huang et al., 2023; Khalil et al., 2021). In conclusion, while moderate consumption is essential, foods rich in SFAs—such as yak milk—should not be entirely avoided, as their health impacts are more complex and context-dependent than previously assumed.

In addition to SFAs, the function of unsaturated fatty acids (UFAs) in milk is also important. Many studies have revealed that yak milk contains an abundance of MUFAs and PUFAs (Pal et al., 2024; Yang et al., 2022), but lipidomics analysis of lipid composition by extracting MFGs is rare. In this study, except for CD3 and Hex3Cer, the concentrations of MUFAs and PUFAs in TG, ChE, DG, Cer, CerP, PE, PC, PI, PIP, PIP2, PS, SM, CL, LPI, Co, and phSM in Y were significantly higher than those in GS (Fig.S12 and S13). Among 187 SDLs, the concentrations of 153 UFAs in Y were significantly higher than those in GS (Table S5, *P*<0.05), including 130 TGs (18:0_18:1_20:1, 18:1_18:3_18:3, 4:0_6:0_18:3, 18:1_18:1_22:5 16:1_14:1_18:2, 18:0_6:0_20:4, and 15:0_18:2_18:3, etc.), 6 DGs (42:5e, 40:6e, 20:2e, 26:2e, 38:6e, and 32:3e), 4 ZyEs (37:6, 35:6, 36:6, and 35:5), 3 ChEs (35:6, 33:6, and 24:7), 3 PSs (18:0_18:1, 18:0_20:4, and 18:1_18:1), 3 PSs (18:0_18:1, 18:0_20:4, and 18:1_18:1), 2 Cers (d18:1_23:0 and d18:1_24:0), 1 CerP (d39:1), and 1 PE (18:1_18:2). It can be seen that Y was richer in UFAs than GS. The crucial aspect is that humans are unable to synthesize certain UFAs such as LA, LNA, EPA, and DHA, so they must obtain these essential fatty acids via their daily diets to maintain lipid balance, facilitate brain and visual development, strengthen immune function, and lower the risk of cancer (Khalid et al., 2022; Mielczarek-Puta et al., 2019). To a certain extent, this can explain why yak milk provides protection for the survival and development of calves and herders in plateau habitats.

### 3.7 Screening and validation of biomarkers between Y and GS

**Fig. 7:**
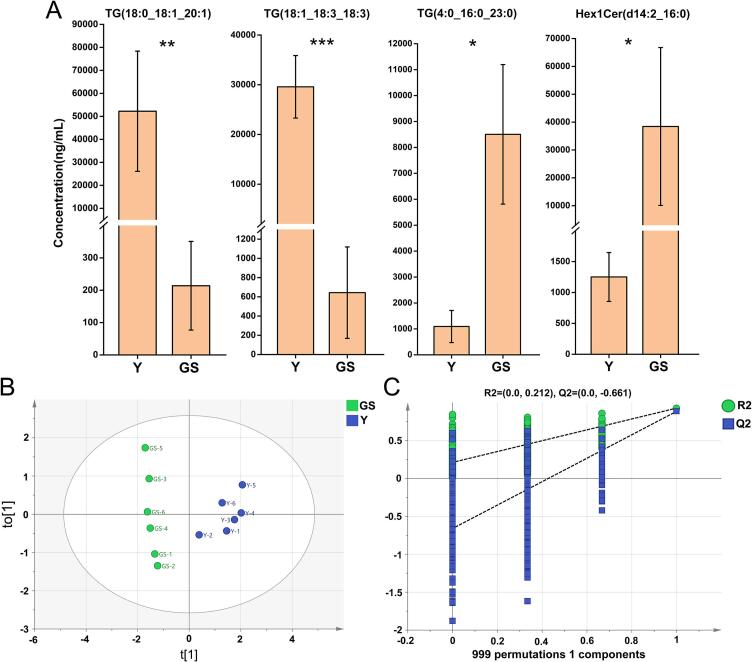
Comparison of levels of four potential biomarkers between Y and GS (A); Plot of OPLS-DA scores for potential biomarkers (B) and the corresponding 999 iterations of permutation tests (C). Y = Yak milk; GS = German Simmental cattle milk. *n* = 6. * means *P* < 0.05, ** means *P* < 0.01, and *** means *P* < 0.001. (For interpretation of the references to color in this figure legend, the reader is referred to the web version of this article).

The search for potential biomarkers helps precisely identify key differential lipids between Y and GS. The FC values of 187 SDLs were sorted in descending order, and a volcano plot analysis was conducted to screen potential lipid biomarker candidates. To more intuitively visualize the distribution of the four potential lipid biomarkers among all SDLs, they are clearly labeled in Fig. 6B. The highest FC value [log_2_(FC)=7.9320] was observed for TG(18:0_18:1_20:1), followed by [log_2_(FC)=5.5233] for TG(18:1_18:3_18:3). Conversely, Hex1Cer(d14:2_16:0) exhibited the lowest FC value [log₂(FC）=−4.9411], with TG(4:0_16:0_23:0) following [log₂(FC)=−2.9607]. When analyzing the concentration profiles of these four potential biomarkers across the two groups, TG(18:0_18:1_20:1) and TG(18:1_18:3_18:3) were significantly more abundant in Y (*P* < 0.01) (Fig. 7A), whereas Hex1Cer(d14:2_16:0) and TG(4:0_16:0_23:0) were significantly more abundant in GS (*P* < 0.05), which were consistent with the change trend of FC values. Subsequently, an OPLS-DA model was constructed using the concentrations of these candidate biomarkers to differentiate between Y and GS. In the single-component OPLS-DA model, distinct separation between Y and GS was evident (Fig. 7B). The t1 values in Y were overall positive, while the t1 values in GS were all in the negative range (Fig. 7B).

Furthermore, 999-iteration permutation tests were performed on the OPLS-DA model. The results showed that the model had satisfactory goodness of fit and prediction accuracy, with R^2^ = 0.212 and Q^2^ = −0.661 (Fig. 7C). This approach aligns with previous studies that systematically selected potential lipid biomarkers from bovine colostrum and milk (Li et al., 2021), human and ewe colostrum (Wang et al., 2023), and yak colostrum and mature milk (Li et al., 2024). PE(P-16:0/20:4), PE(P-16:0/22:5), Hex2Cer(d15:0/24:1), and Hex2Cer(d14:0/24:0) were selected as potential biomarkers for discriminant analysis of bovine colostrum and milk (Li et al., 2021). Similarly, TG(10:0/15:0/16:0), TG(18:1/24:1/18:2), DG(19:0/18:0), and FFA(22:0) served as key biomarkers to differentiate lipid profiles between human and ewe colostrum (Wang et al., 2023). In addition, four potential lipid biomarkers including 1 CE(16:0) and 3 TAGs ((54:6 (20:5), 50:1 (16:0), and 56:6 (20:5)) were selected to provide theoretical reference for the identification and detection of yak colostrum and mature milk (Li et al., 2024). In conclusion, these results laid a theoretical foundation for confirming that four lipid biomarkers are applicable in differentiating between yak mature milk and German Simmental cattle mature milk, which further provided a theoretical reference for the identification of real and fake yak milk in the market.

### 3.8 Correlation analysis of SDLs between Y and GS

**Fig. 8:**
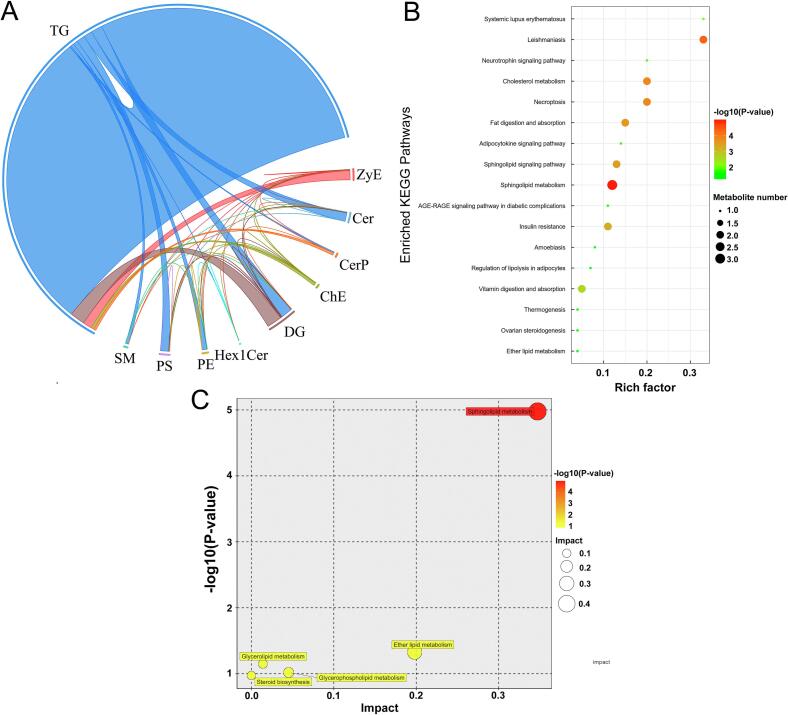
A chord diagram of lipid-lipid correlation of 187 SDLs between Y and GS (A). KEGG pathway analysis of 187 SDLs between Y and GS (B). Metabolomic view map of important lipid metabolic pathways of 187 SDLs between Y and GS (C). The X-axis represents the pathway impact, and the Y-axis represents the -log_10_(*P*-value). Large sizes and dark colors represent high pathway impact values and major pathway enrichment, respectively. Y = Yak milk; GS = German Simmental cattle milk; SDLs: significantly different lipids.

To further quantify metabolic proximity and visualize relationships among 187 SDLs identified between Y and GS, a correlation analysis was performed based on lipid structures and physiological property similarities in this study (Fig. S14). Heatmap analysis of the 187 SDLs revealed that most exhibited significant positive correlations, while only 8 SDLs (TG(18:0_16:0_16:0), Hex1Cer(t42:2), Hex1Cer(d21:2_19:0+O), TG(12:0e_6:0_18:4), TG(20:1e_6:0_6:0), TG(32:3e), TG(4:0_16:0_23:0), and Hex1Cer(d14:2_16:0)) showed significant negative correlations with other lipids (Fig.S14 and Table S7). Among these, 5 pairs of SDLs, TG (18:0 _16:0 _18:3) and TG (15:0 _6:0 _18:2), ChE(35:6) and ZyE(35:5), TG (18:0 _18:1 _23:1) and TG (18:0 _18:1 _23:0), TG (18:0 _18:1 _23:0) and TG (18:0 _17:0 _23:0), and DG(32:3e) and DG(30:0e), had the highest correlation coefficients (*P*<0.001, r>0.998) (Table S7). Chord and network diagram analyses were conducted for the 187 SDLs with correlation coefficients |r| > 0.8 and *P* < 0.05 to more intuitively reveal their co-regulatory relationships (Fig. 8A and Table S7). The 160 TGs were intercorrelated and also highly correlated with DGs, ZyEs, PSs, ChEs, Hex1Cers, Cers, PEs, CerP, and SM (Fig. 8A). Moreover, most SDLs had a high degree of correlation (167 of 187 SDLs with a degree value >100), and correlations within single lipid classes were stronger than those between different classes (Table S8). Notably, in both Y and GS, TGs, DGs, and Cers formed intricate relationship clusters with other SDL subclasses, indicating that glycerolipids and sphingolipids were not only homeostatically regulated but also served as connectors for interconversion with glycerophospholipids (PE and PS), sterol lipids (ChE and ZyE), and other sphingolipids (Hex1Cer, CerP, and SM). This finding also further strengthened the view that GP was a major substrate and intermediate in lipid biosynthesis (Jha et al., 2018; Lee & Ridgway, 2020). Collectively, our results indicate close correlations among lipid species, such that alterations in any one species could potentially affect the levels of numerous other lipid subclasses during lactation. Similar results were observed in lipid characterizations comparing bovine colostrum and mature milk, human colostrum and ewe colostrum, and yak colostrum and mature milk (Li et al., 2020, Li et al., 2024, Wang et al., 2023).

### 3.9 Metabolic pathway enrichment analysis of SDLs

To clarify the changes in metabolic pathways of yak milk and German Simmental cattle milk lipids, 187 SDLs were mapped to KEGG databases (Table S9). A total of 23 KEGG metabolic pathways incorporated these SDLs, and notably, 17 of them were significantly enriched (*P* < 0.05), including sphingolipid metabolism, sphingolipid signaling pathway, fat digestion and absorption, cholesterol metabolism, vitamin digestion and absorption, and ether lipid metabolism (Fig. 8B and Table S9). KEGG enrichment analysis revealed disparities in lipid composition and function between the two mammalian species. To further understand the differences in lipid metabolic pathways between Y and GS, 187 SDLs were input into MetaboAnalyst 6.0 to overview important lipid metabolic pathways (Fig. 8C). Sphingolipid metabolism appeared with the largest bubble and darkest color (*P* < 0.0001), indicating it was the most essential metabolic pathway between Y and GS. This was followed by ether lipid metabolism (*P* < 0.05), glycerolipid metabolism, glycerophospholipid metabolism, and steroid biosynthesis, but the last three pathways showed no significance (*P* > 0.05). Notably, 173 SDLs identified between human and ewe colostrum (Wang et al., 2023) were mainly enriched in the sphingolipid metabolism pathway, consistent with our results. However, 43 SDLs identified between yak colostrum and mature milk (Li et al., 2024), and 63 SDLs between bovine colostrum and mature milk (Li et al., 2020), were primarily concentrated in the glycerophospholipid metabolic pathway, followed by steroid biosynthesis and sphingolipid metabolism (Li et al., 2024; Li et al., 2020), which differed from this study. Previous research has shown that yak milk contains a unique sphingolipid composition and abundant complex glycosphingolipid structures (Qu et al., 2016), which may explain the significant enrichment of these SDLs in the sphingolipid metabolism pathway.

Sphingolipids, ubiquitous constituents of biological membranes across all eukaryotic organisms, play a pivotal role in signal transduction pathways. Moreover, they are directly involved in essential biological processes, including cell proliferation, growth, differentiation, aging, migration, and apoptosis (Iessi et al., 2020; Quinville et al., 2021). Previous studies have shown that sphingosine-1-phosphate promotes cell proliferation, survival, and differentiation (Lu et al., 2015), whereas ceramide induces cell apoptosis and autophagy (Alizadeh et al., 2023). The sphingolipid metabolic pathway exhibits an complex network of reactions that leads to the formation of multiple sphingolipids, such as ceramide, sphingosine, and sphingosine-1-phosphate (Hannun & Obeid, 2018). Additionally, together with cholesterol and glycolipids, sphingolipids (mainly sphingomyelin) constitute one of the fundamental lipid components of lipid rafts, and cholesterol- and sphingomyelin-enriched membrane microdomains are directly implicated in cell survival and death mechanisms (D'Aprile et al., 2021; Iessi et al., 2020). Furthermore, both cholesterol metabolism and vitamin digestion/absorption have emerged as potential pathways for regulating the number of carbon atoms and double bonds in triacylglycerol (Zhang et al., 2022). The pathway analysis of SDLs between Y and GS will not only facilitate deeper exploration of the biological functions of these SDLs but also enhance our understanding of the lipid profiles in the mature milk of the two mammalian species.

## 4 Conclusions

In this study, the composition and MFGs size of mature milk from yaks and German Simmental cattle were determined, and these extracted MFGs in milk were characterized by the absolute quantitative lipidomics approach. The milk fat, lactoprotein, lactose, SNF, salt and ice point in Y were significantly higher than those in GS. Compared with GS, the proportion of large MFGs in Y was higher. Overall, 2414 lipid species from 43 lipid subclasses were identified in Y and GS, and 187 SDLs from 10 lipid subclasses were subsequently screened. Four potential lipid biomarkers, namely, TG(18:0_18:1_20:1) and TG(18:1_18:3_18:3), Hex1Cer(d14:2_16:0) and TG(4:0_16:0_23:0), were identified. Furthermore, yak mature milk had higher proportion of short−/medium−/long-chain SFAs, MUFAs and PUFAs than German Simmental cattle mature milk. Most of the 187 SDLs were significantly positively correlated, with only 8 showing significant negative correlations with other SDLs. These 187 SDLs were involved in 23 KEGG metabolic pathways, of which 17 were significantly enriched, and sphingolipid metabolism were the most relevant. These identified SDLs provided an in-depth understanding of lipid compositions in yak and German Simmental cattle milk, clarifying differences in their mature milk lipid profiles and offering practical information for yak milk product development. They also helped evaluate the authenticity and quality of yak mature milk and understand the biological activity of its lipids. Future research will focus on larger samples to confirm the value of screened lipid biomarkers and guide the development of precise nutritional formulations from yak milk.

## CRediT authorship contribution statement

**Yili Liu:** Writing – original draft, Visualization, Methodology. **Peng Wang:** Supervision, Resources, Formal analysis. **Kai Wang:** Visualization, Resources, Formal analysis, Data curation. **Yueyue Li:** Visualization, Investigation. **Zifeng Ma:** Validation, Methodology. **Jian Li:** Validation, Methodology. **Daoliang Lan:** Validation, Investigation. **Liqiang Han:** Validation, Investigation. **Wei Fu:** Resources, Project administration, Funding acquisition, Conceptualization.

## Declaration of competing interest

The authors declare that they have no known competing financial interests or personal relationships that could have appeared to influence the work reported in this paper.

## Data Availability

Data will be made available on request.

## Appendix A Supplementary data

Supplementary material 1 Image 1

Supplementary material 2 Image 2

Supplementary material 3 Image 3

Supplementary material 4 Image 4

Supplementary material 5 Image 5

Supplementary material 6 Image 6

Supplementary material 7 Image 7

Supplementary material 8 Image 8

Supplementary material 9 Image 9

Supplementary material 10 Image 10
