# Supplementary material for: Absolute quantitative Lipidomics reveals lipid differences in milk fat globules of yak and German Simmental cattle
Source: Food Chem X. 2025 Jun 23;29:102686. doi: 10.1016/j.fochx.2025.102686 (PMC12270805; doi:10.1016/j.fochx.2025.102686)
Supplement: Supplementary file 1 — Supplementary material 1 [file mmc1.docx]

**Figure and table captions**

**Fig.S1.** Determination of milk salt (A) and freezing point (B) in Y and GS.

**Fig. S2.** Base Peak Chromatograms (BPC) overlap map of QC samples in positive and negative ion mode. Positive ion mode (A). Negative ion mode (B). QC= quality control.

**Fig.S3.** Repeatability of experiment and reliability of the analytical methods. Correlation map of QC samples (A). The x-axis and y-axis represent the logarithmic values of ion peak signal intensity. PCA scores plot of all samples (B). 3D-PCA score plot of all samples (C). Hotelling’s T^2^ plot for all samples (D). MCC diagram of QC samples (E). The x-axis represents the various QC samples, and the horizontal coordinate represents each QC sample, the vertical coordinate reflects the standard deviation, and the yellow and red lines define the range of plus or minus 2 and 3 standard deviations respectively. Relative standard deviation of QC samples (F). QC= quality control; Y=yak milk; GS=German Simmental cattle milk.

**Fig.S4**. Composition of lipid subclasses in Y and GS. Y=yak milk; GS=German Simmental milk.

**Fig.S5.** Dynamic distribution range of lipid content in Y and GS. Y=yak milk; GS=German Simmental cattle milk.

**Fig.S6.** The top 20 enriched KEGG pathways of lipids in Y and GS. Y=yak milk; GS=German Simmental cattle milk.

**Fig.S7.** Comparison of low concentration lipid subclasses with significant differences between Y and GS. Y=yak milk; GS=German Simmental cattle milk.

**Fig.S8**. Comparison of low concentration lipid subclasses with significant differences between Y and GS. Y=yak milk; GS=German Simmental cattle milk.

**Fig.S9.** Chain Length Analysis of TGs.

**Fig.S10**. Chain saturation analysis of TGs.

**Fig.S11.** Saturated fatty acids significantly upregulated in Y compared to GS. Y=yak milk; GS=German Simmental cattle milk.

**Fig.S12.** Unsaturated fatty acids significantly upregulated in Y compared to GS. Y=yak milk; GS=German Simmental cattle milk.

**Fig.S13.** Unsaturated fatty acids significantly downregulated in Y compared to GS. Y=yak milk; GS=German Simmental cattle milk.

**Fig.S14.** The correlation analysis between 187 significantly different lipids.

**Table S1.** The information of top 20 lipids molecules in Y and GS.

**Table S2.** KEGG pathway analysis of lipids in Y and GS.

**Table S3.** Lipid subclasses with significant differences in content.

**Table S4.** Differential lipid molecules based on T-test analysis between Y and GS.

**Table S5.** The significantly different lipids with VIP＞1 and *P*＜0.05.

**Table S6.** Analysis of chain length and chain saturation of 160 significantly different TG species.

**Table S7.** The correlation analysis between 187 SDLs（r＞0.8 and *P*＜0.05）.

**Table S8.** The degree of 187 SDLs.

**Table S9.** KEGG pathway analysis of 187 SDLs.


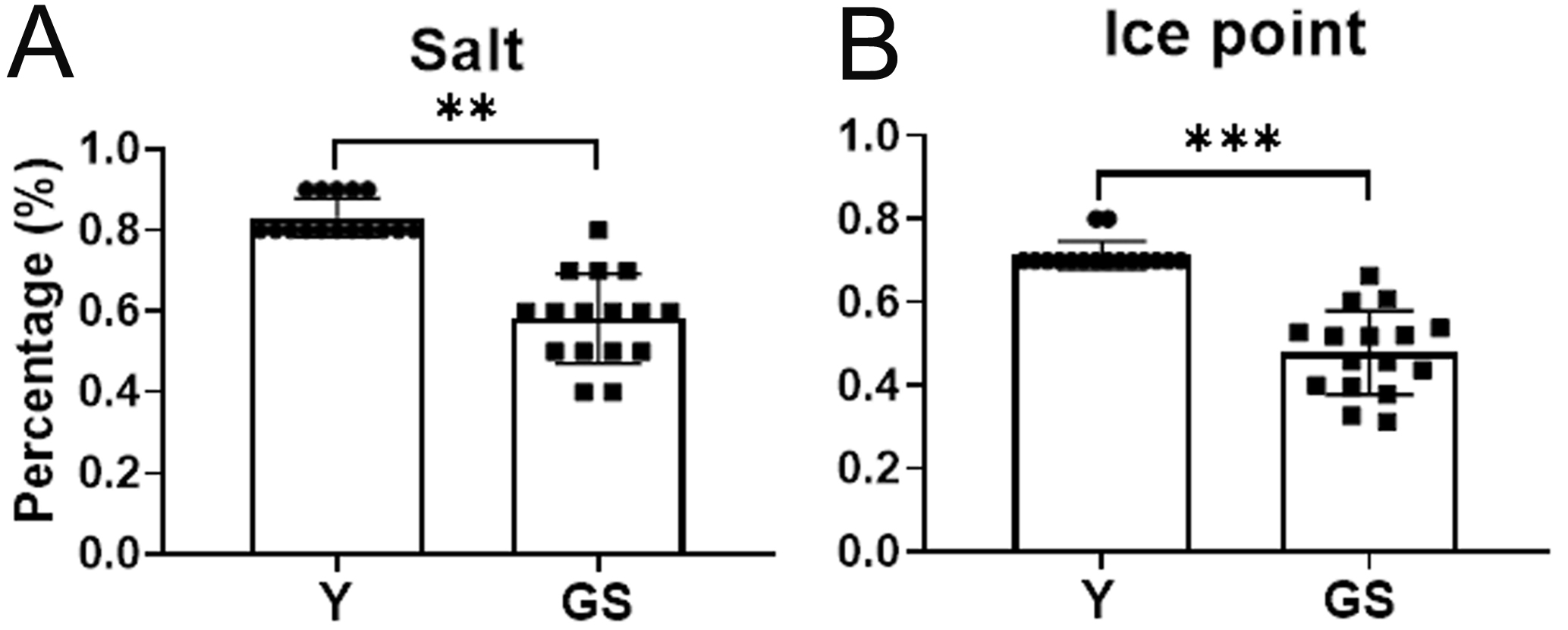


**Fig.S1.** Determination of milk salt (A) and freezing point (B) in Y and GS.


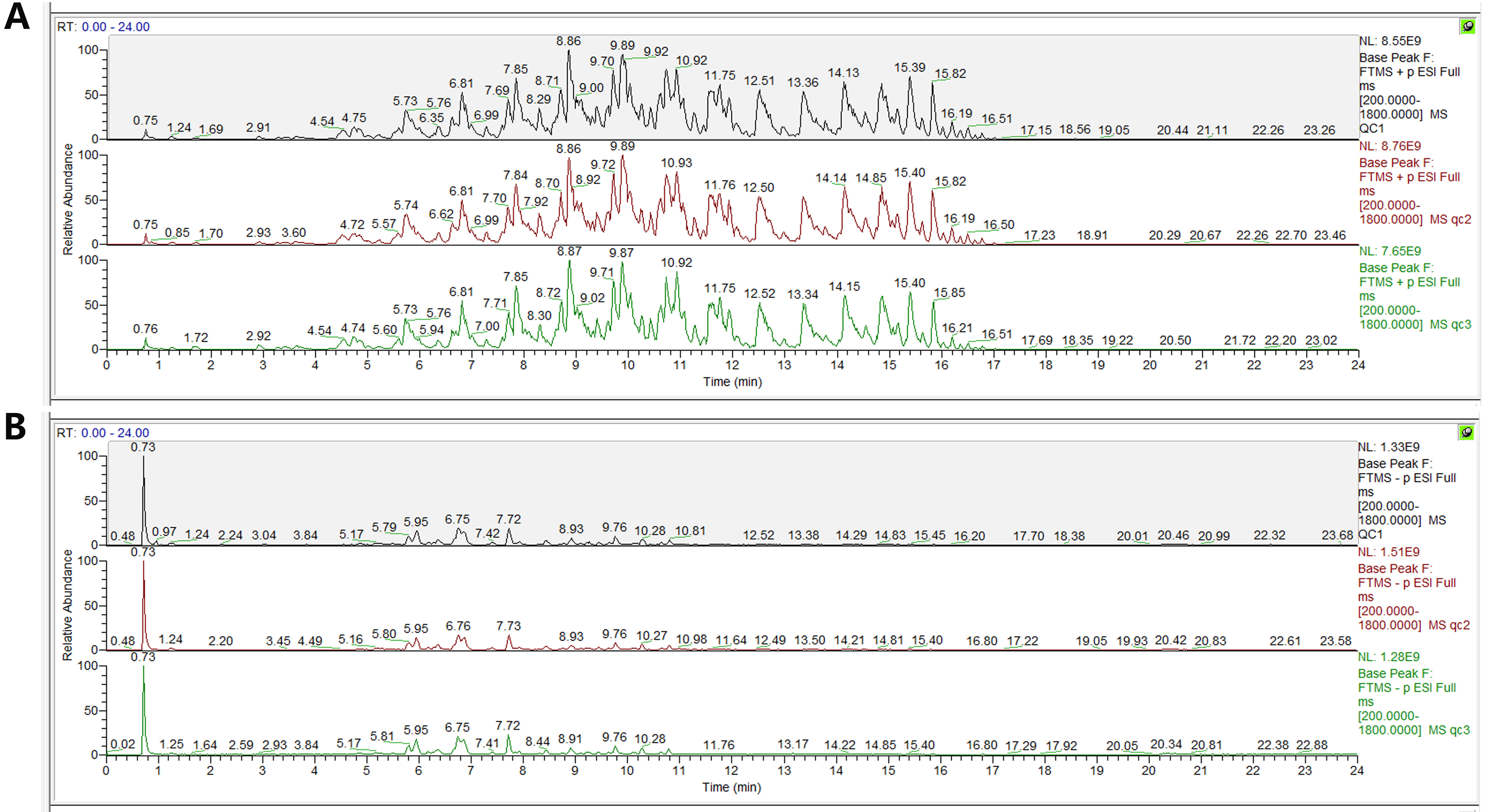


**Fig. S2.** Base Peak Chromatograms (BPC) overlap map of QC samples in positive ion (A) and negative ion (B) mode. QC= quality control. The horizontal coordinate represents the retention time of each chromatographic peak, and the vertical coordinate represents the intensity value of the peak.

**
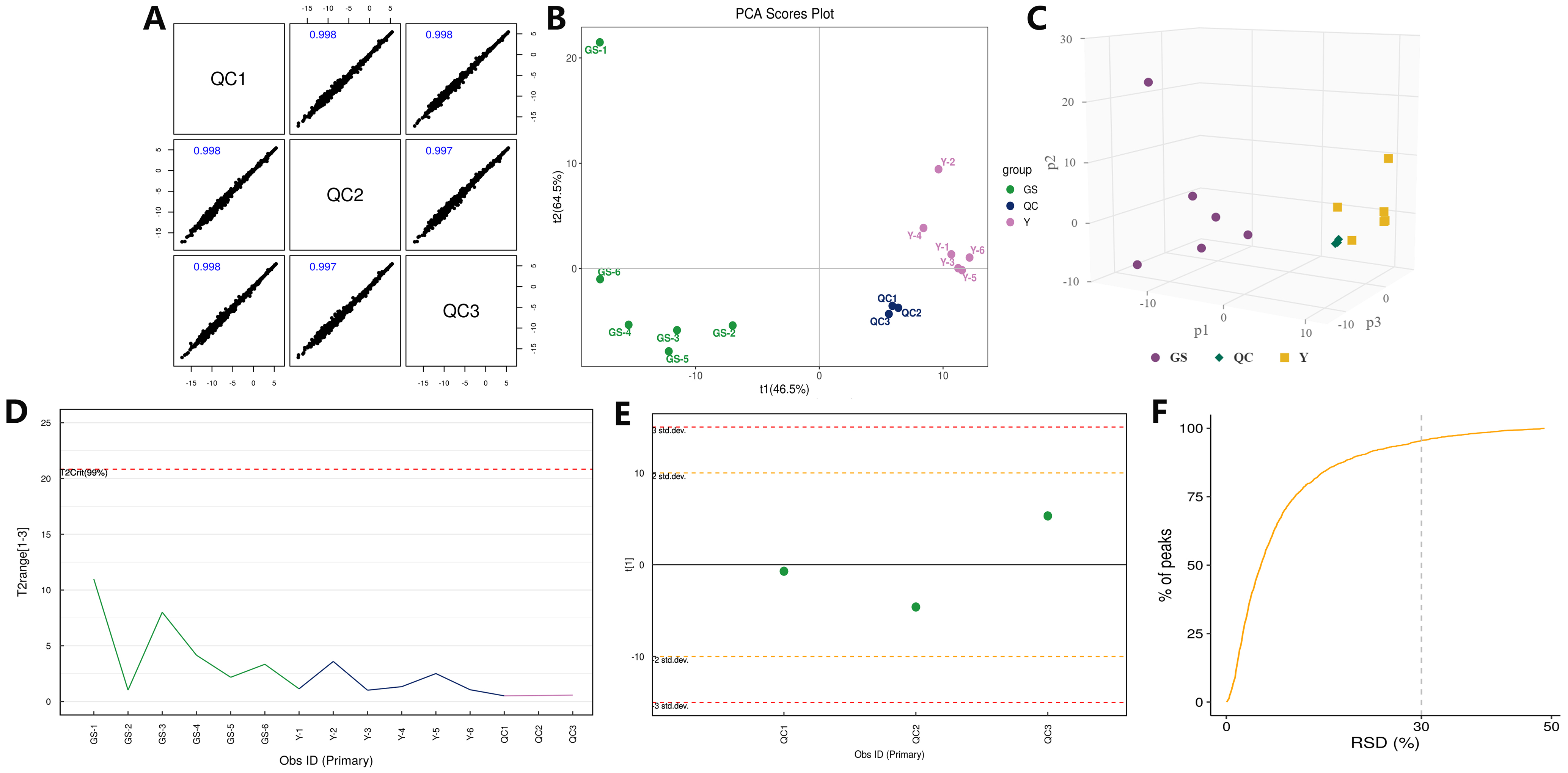
**

**Fig.S3.** Repeatability of experiment and reliability of the analytical methods. Correlation map of QC samples. The x-axis and y-axis represent the logarithmic values of ion peak signal intensity (A). PCA scores plot of all samples (B). 3D-PCA score plot of all samples (C). Hotelling’s T^2^ plot for all samples (D). MCC diagram of QC samples (E). The x-axis represents the various QC samples, and the horizontal coordinate represents each QC sample, the vertical coordinate reflects the standard deviation, and the yellow and red lines define the range of plus or minus 2 and 3 standard deviations respectively. Relative standard deviation of QC samples (F). QC= quality control; Y=yak milk; GS=German Simmental milk.


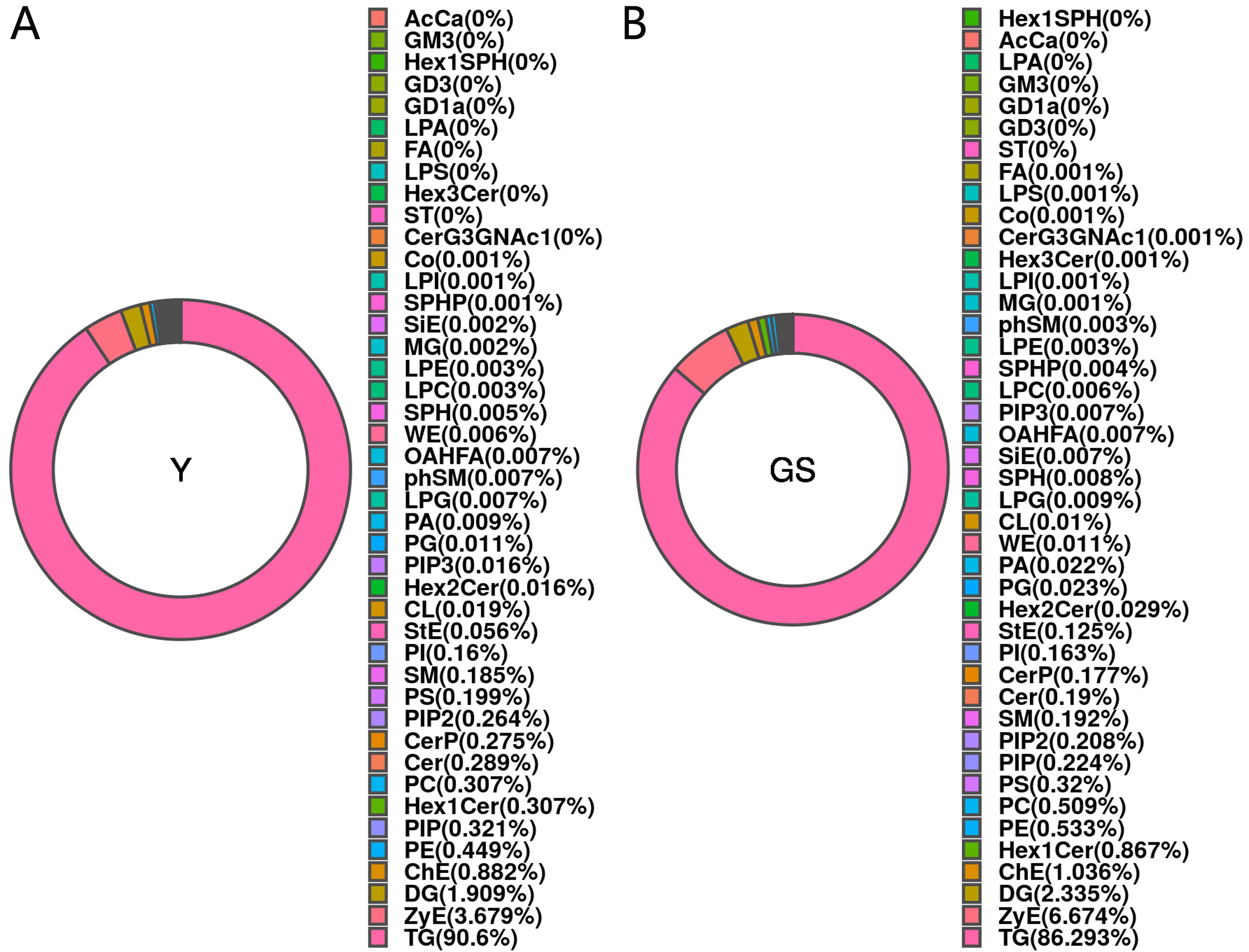


**Fig.S4**. Composition of lipid subclasses in Y and GS.Y=yak milk; GS=German Simmental milk.


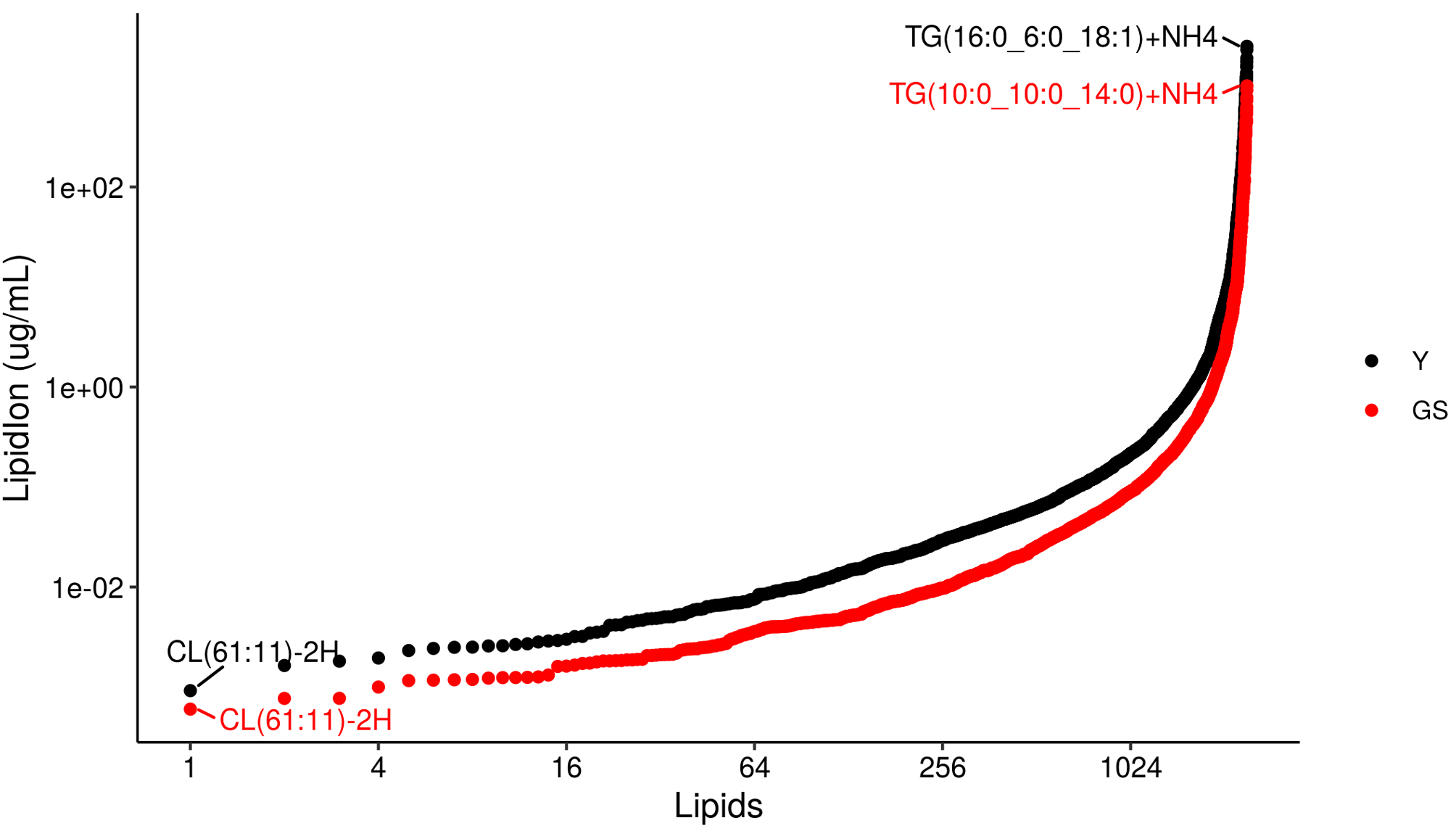


**Fig.S5.** Dynamic distribution range of lipid content in Y and GS. Y=yak milk; GS=German Simmental cattle milk.


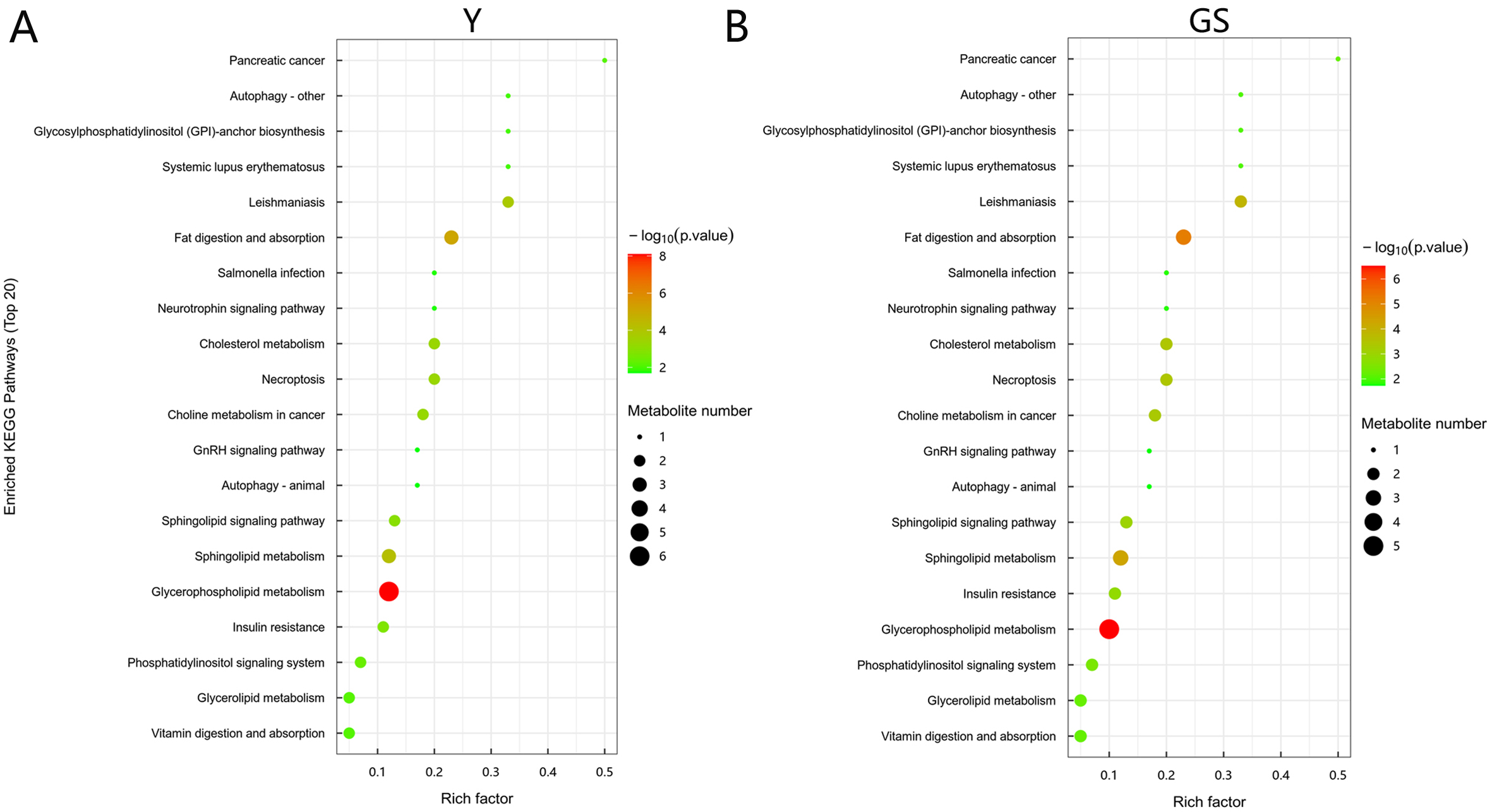


**Fig.S6.** The top 20 enriched KEGG pathways of lipids in Y and GS. Y=yak milk; GS=German Simmental milk.


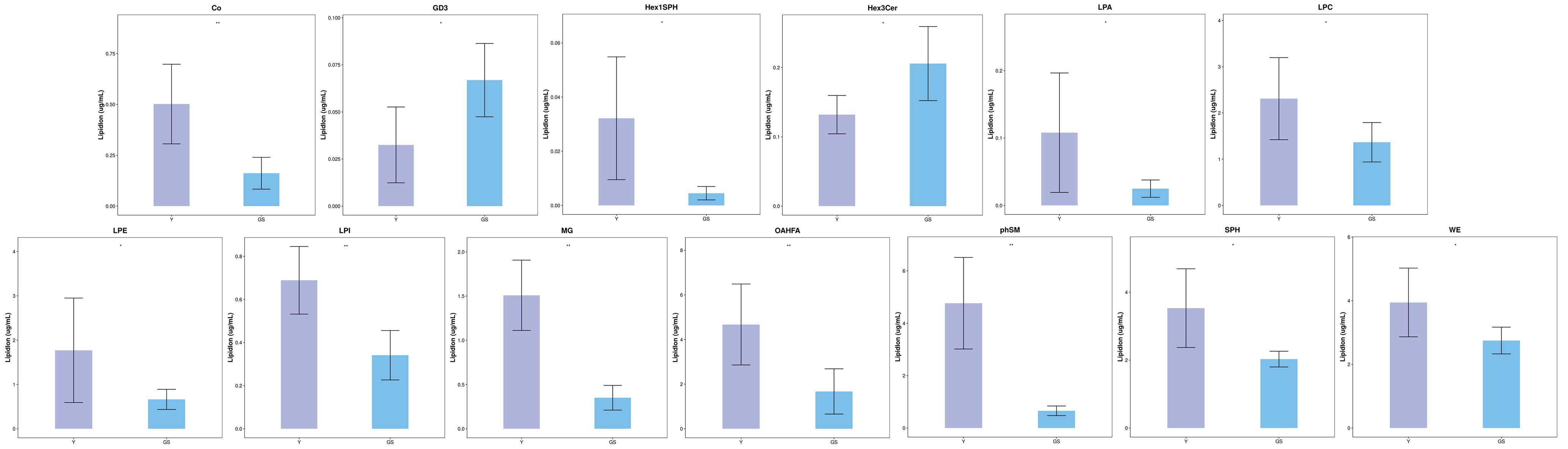
**Fig.S7.** Comparison of low concentration lipid subclasses with significant differences between Y and GS. Y=yak milk; GS=German Simmental cattle milk.


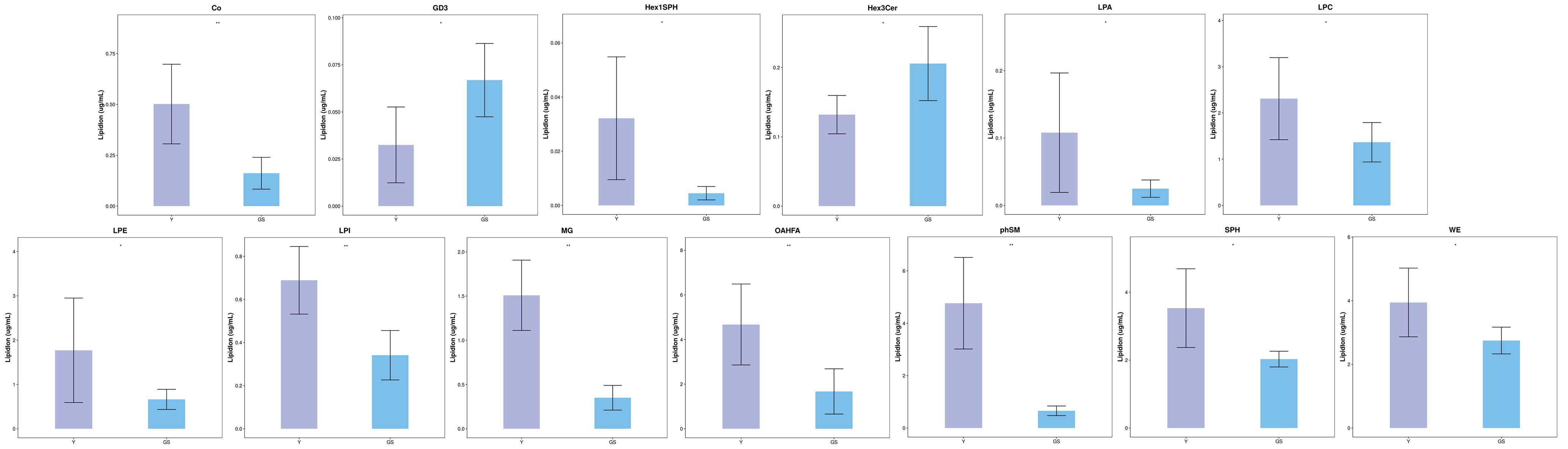


**Fig.S8**. Comparison of low concentration lipid subclasses with significant differences between Y and GS. Y=yak milk; GS=German Simmental cattle milk.


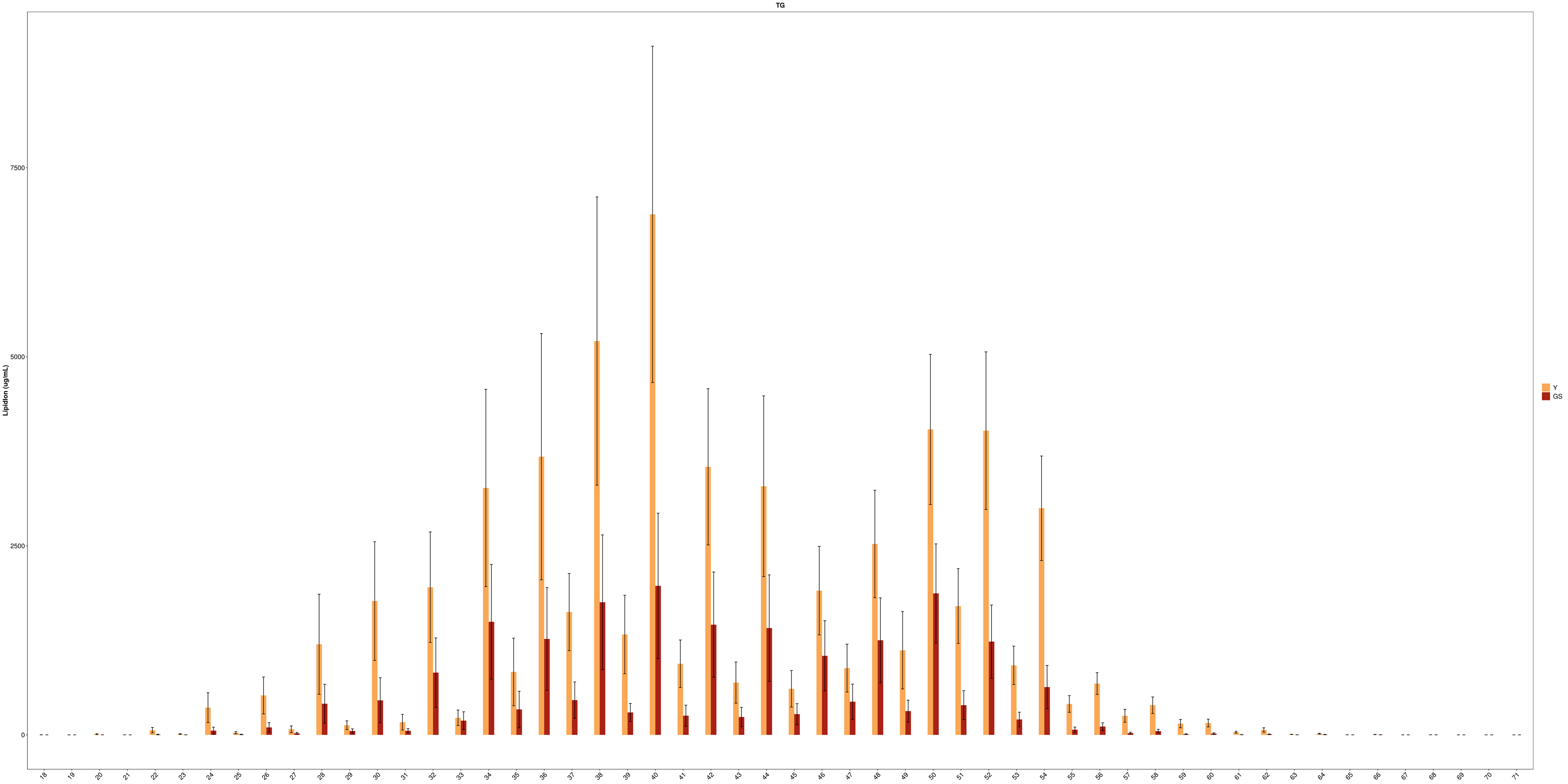


**Fig.S9**. Chain Length Analysis of TGs.


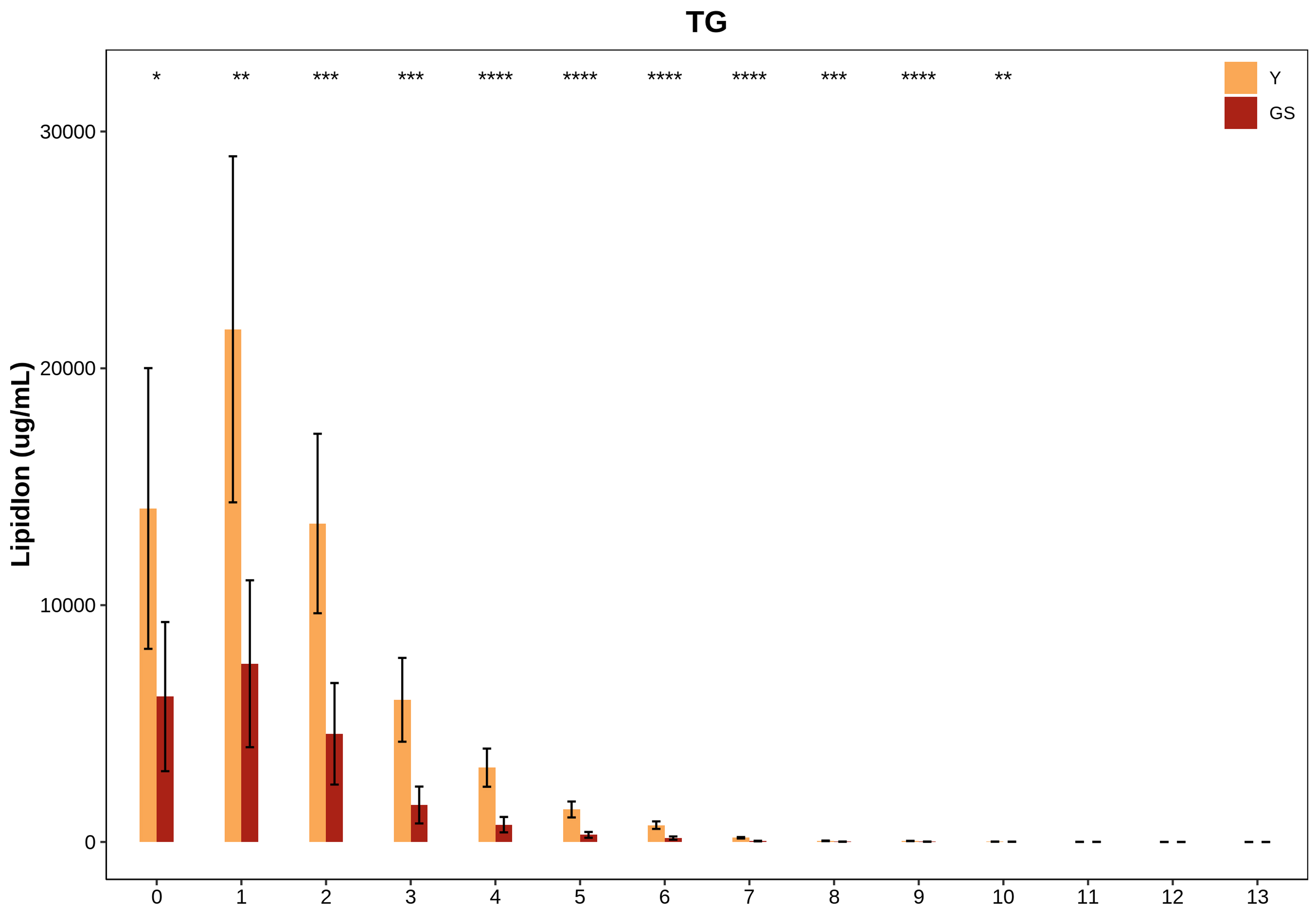


**Fig.S10**. Chain saturation analysis of TGs.


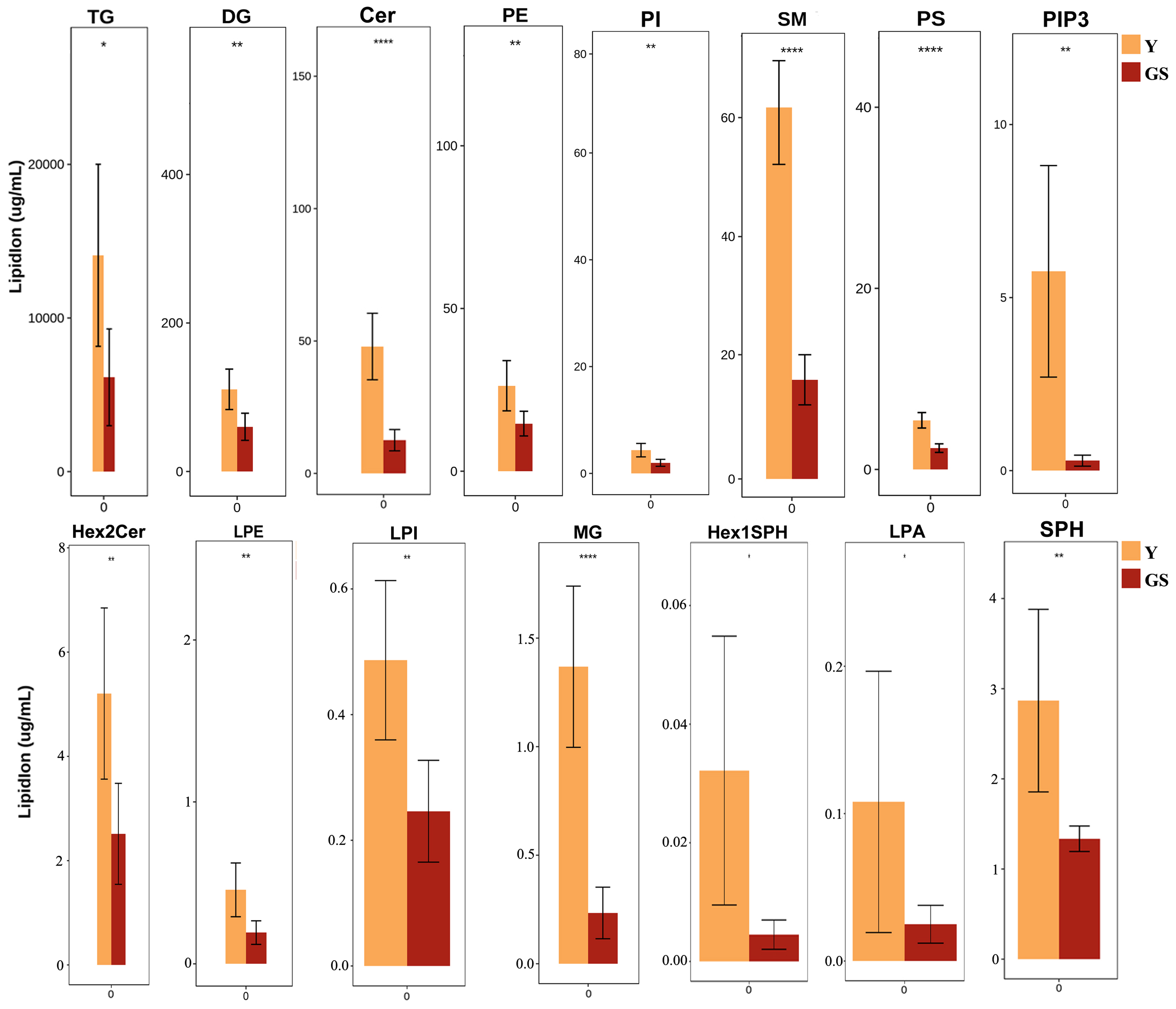


**Fig.S11.** Saturated fatty acids significantly upregulated in Y compared to GS. Y=yak milk; GS=German Simmental cattle milk.


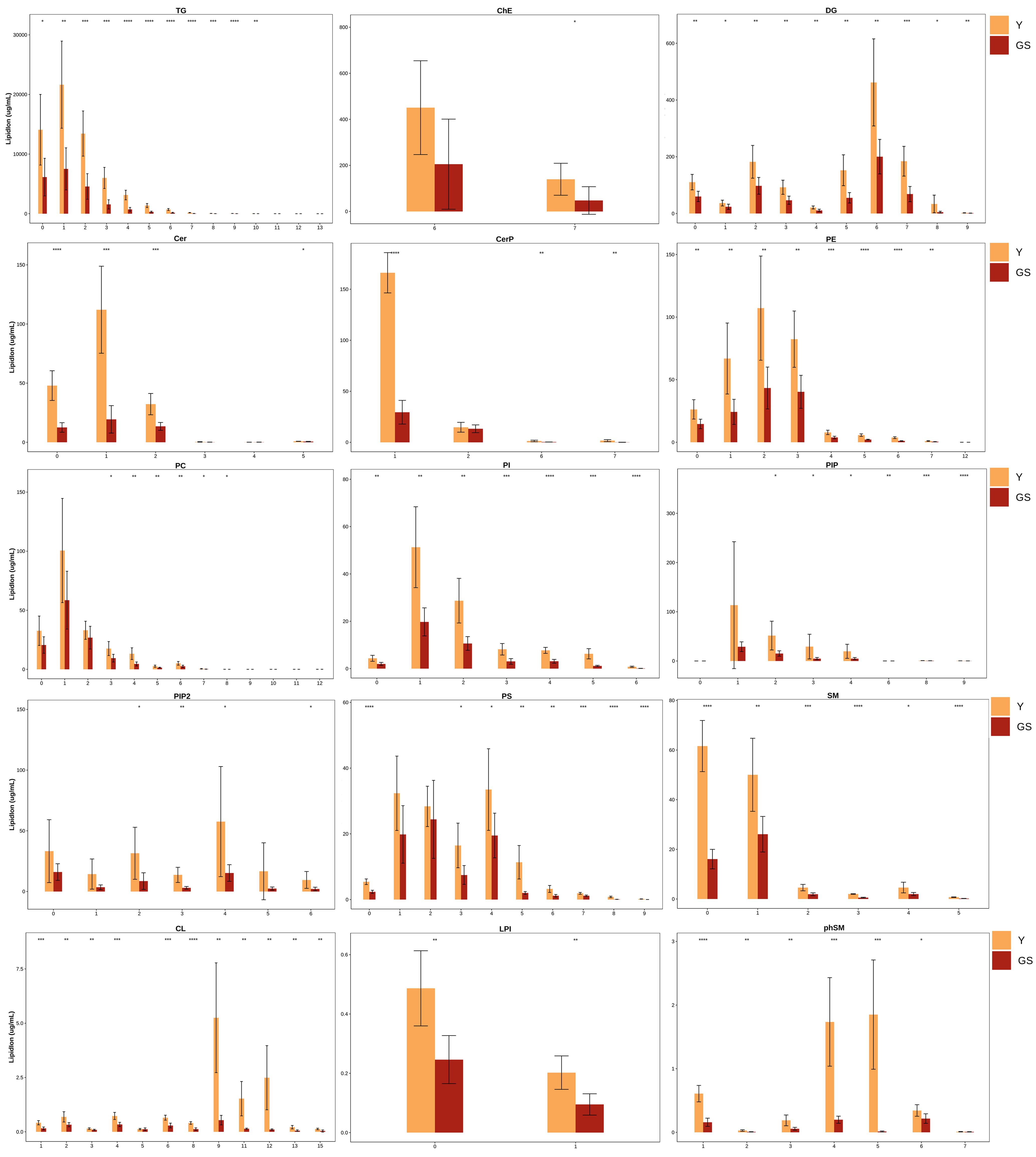


**Fig.S12.** Unsaturated fatty acids significantly upregulated in Y compared to GS. Y=yak milk; GS=German Simmental cattle milk.


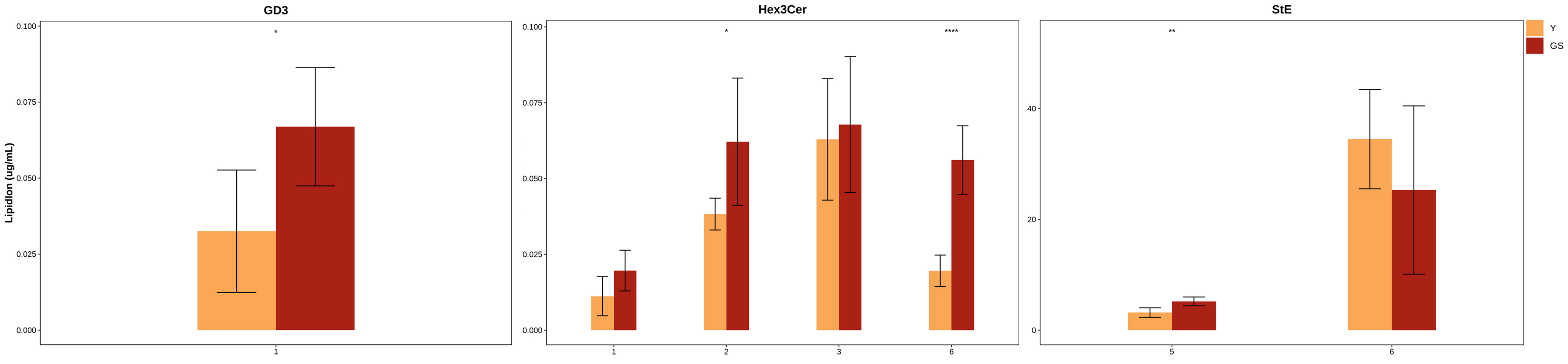


**Fig.S13.** Unsaturated fatty acids significantly downregulated in Y compared to GS. Y=yak milk; GS=German Simmental cattle milk.


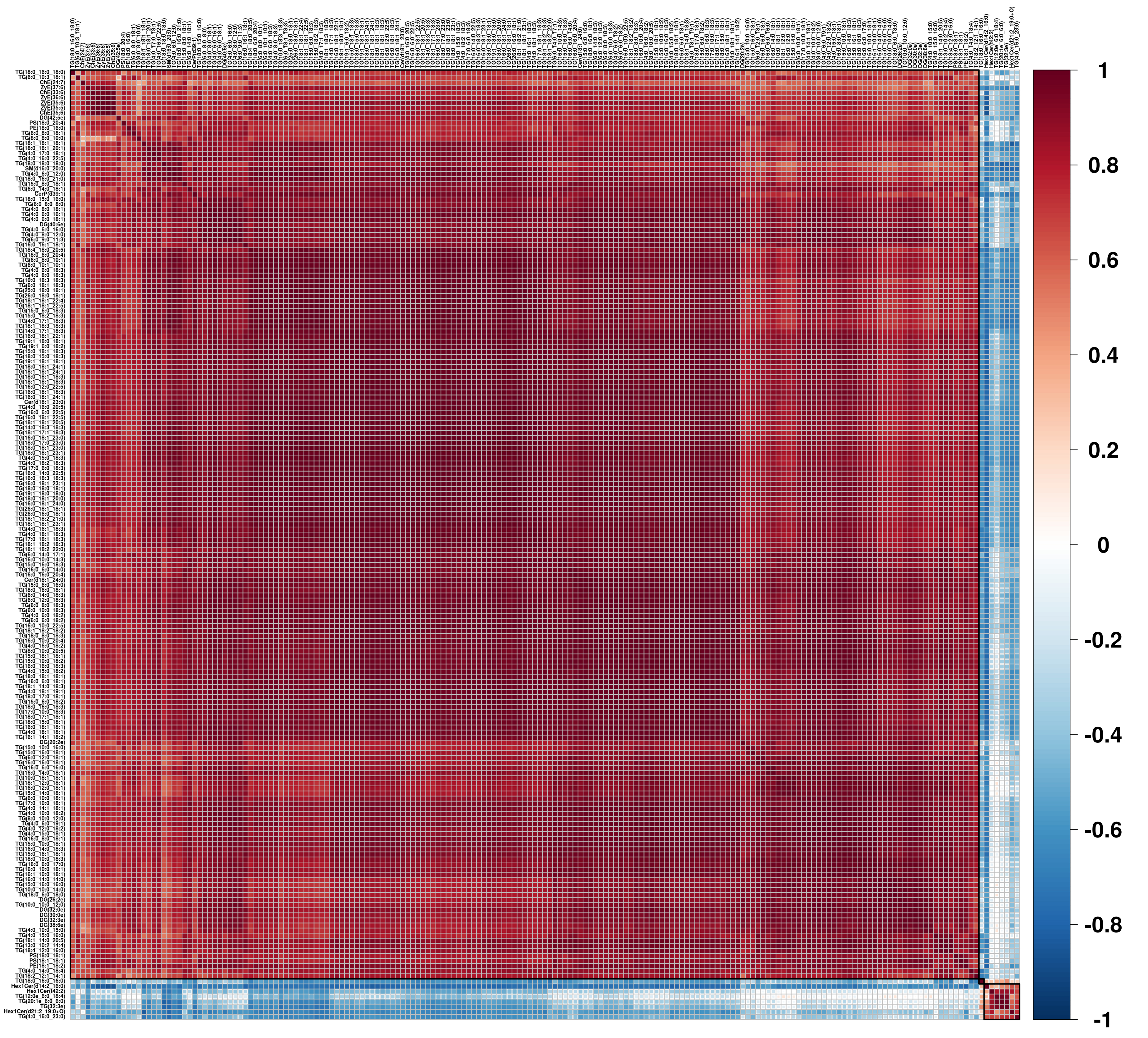


**Fig.S14.** The correlation analysis between 187 significantly different lipids.
