# Supplementary material for: Absolute quantitative Lipidomics reveals lipid differences in milk fat globules of yak and German Simmental cattle
Source: Food Chem X. 2025 Jun 23;29:102686. doi: 10.1016/j.fochx.2025.102686 (PMC12270805; doi:10.1016/j.fochx.2025.102686)
Supplement: Supplementary file 2 — Supplementary material 2 [file mmc2.docx]

**Table S1**. The information of top 20 lipids molecules in Y and GS

| **Group** | **name** | **Lipid Ion** | **Lipid Group** | **Class** | **Fatty Acid** | **FA1** | **FA2** | **FA3** | **Fold Change (Y/GS)** | ***P*-value** | **VIP** |
| --- | --- | --- | --- | --- | --- | --- | --- | --- | --- | --- | --- |
| Y | POS3351 | TG(16:0_6:0_18:1)+NH4 | TG(40:1)+NH4 | TG | (16:0_6:0_18:1) | (16:0) | (6:0) | (18:1) | 4.292457437 | 0.000264 | 10.14785 |
| Y | POS3112 | TG(6:0_14:0_18:1)+NH4 | TG(38:1)+NH4 | TG | (6:0_14:0_18:1) | (6:0) | (14:0) | (18:1) | 2.532557512 | 0.004047 | 4.938542 |
| Y | POS2682 | TG(10:0_10:0_14:0)+NH4 | TG(34:0)+NH4 | TG | (10:0_10:0_14:0) | (10:0) | (10:0) | (14:0) | 1.890094753 | 0.032665 | 9.706381 |
| Y | POS4424 | TG(16:0_16:1_18:1)+NH4 | TG(50:2)+NH4 | TG | (16:0_16:1_18:1) | (16:0) | (16:1) | (18:1) | 2.283823516 | 0.000681 | 3.934822 |
| Y | POS2907 | TG(6:0_12:0_18:1)+NH4 | TG(36:1)+NH4 | TG | (6:0_12:0_18:1) | (6:0) | (12:0) | (18:1) | 2.204465775 | 0.020674 | 5.378025 |
| Y | POS3597 | TG(16:0_8:0_18:1)+NH4 | TG(42:1)+NH4 | TG | (16:0_8:0_18:1) | (16:0) | (8:0) | (18:1) | 2.542692643 | 0.002342 | 3.376033 |
| Y | POS3390 | TG(4:0_18:1_18:1)+NH4 | TG(40:2)+NH4 | TG | (4:0_18:1_18:1) | (4:0) | (18:1) | (18:1) | 3.205109061 | 0.000265 | 4.086534 |
| Y | POS3057 | TG(16:0_6:0_16:0)+NH4 | TG(38:0)+NH4 | TG | (16:0_6:0_16:0) | (16:0) | (6:0) | (16:0) | 3.645751259 | 0.012446 | 4.664742 |
| Y | POS3403 | TG(16:0_10:0_14:3)+NH4 | TG(40:3)+NH4 | TG | (16:0_10:0_14:3) | (16:0) | (10:0) | (14:3) | 3.813932693 | 0.000482 | 5.863239 |
| Y | POS4663 | TG(16:0_18:1_18:1)+NH4 | TG(52:2)+NH4 | TG | (16:0_18:1_18:1) | (16:0) | (18:1) | (18:1) | 3.021477302 | 0.000662 | 1.713745 |
| Y | POS4177 | TG(16:0_14:0_18:1)+NH4 | TG(48:1)+NH4 | TG | (16:0_14:0_18:1) | (16:0) | (14:0) | (18:1) | 2.273645944 | 0.006085 | 4.095671 |
| Y | POS3790 | TG(16:0_10:0_18:1)+NH4 | TG(44:1)+NH4 | TG | (16:0_10:0_18:1) | (16:0) | (10:0) | (18:1) | 2.242268004 | 0.007968 | 4.836063 |
| Y | POS2424 | TG(8:0_10:0_12:0)+NH4 | TG(30:0)+NH4 | TG | (8:0_10:0_12:0) | (8:0) | (10:0) | (12:0) | 3.297379647 | 0.007678 | 4.47692 |
| Y | POS3156 | TG(4:0_16:0_18:2)+NH4 | TG(38:2)+NH4 | TG | (4:0_16:0_18:2) | (4:0) | (16:0) | (18:2) | 3.263954889 | 0.000512 | 4.549722 |
| Y | POS2858 | TG(16:0_6:0_14:0)+NH4 | TG(36:0)+NH4 | TG | (16:0_6:0_14:0) | (16:0) | (6:0) | (14:0) | 4.768513142 | 0.009565 | 5.889172 |
| Y | POS3821 | TG(16:1_10:0_18:1)+NH4 | TG(44:2)+NH4 | TG | (16:1_10:0_18:1) | (16:1) | (10:0) | (18:1) | 2.361259822 | 0.012281 | 3.682671 |
| Y | POS4376 | TG(16:0_16:0_18:1)+NH4 | TG(50:1)+NH4 | TG | (16:0_16:0_18:1) | (16:0) | (16:0) | (18:1) | 1.840078319 | 0.026722 | 7.380755 |
| Y | POS2528 | TG(10:0_10:0_12:0)+NH4 | TG(32:0)+NH4 | TG | (10:0_10:0_12:0) | (10:0) | (10:0) | (12:0) | 2.083005178 | 0.021925 | 5.30981 |
| Y | POS4604 | TG(18:0_16:0_18:1)+NH4 | TG(52:1)+NH4 | TG | (18:0_16:0_18:1) | (18:0) | (16:0) | (18:1) | 2.729270925 | 0.000425 | 1.031532 |
| Y | POS3542 | TG(18:0_6:0_18:0)+NH4 | TG(42:0)+NH4 | TG | (18:0_6:0_18:0) | (18:0) | (6:0) | (18:0) | 1.839154018 | 0.045852 | 6.629941 |
| GS | POS2682 | TG(10:0_10:0_14:0)+NH4 | TG(34:0)+NH4 | TG | (10:0_10:0_14:0) | (10:0) | (10:0) | (14:0) | 1.890094753 | 0.032665 | 9.706381 |
| GS | POS3112 | TG(6:0_14:0_18:1)+NH4 | TG(38:1)+NH4 | TG | (6:0_14:0_18:1) | (6:0) | (14:0) | (18:1) | 2.532557512 | 0.004047 | 4.938542 |
| GS | POS4424 | TG(16:0_16:1_18:1)+NH4 | TG(50:2)+NH4 | TG | (16:0_16:1_18:1) | (16:0) | (16:1) | (18:1) | 2.283823516 | 0.000681 | 3.934822 |
| GS | POS2907 | TG(6:0_12:0_18:1)+NH4 | TG(36:1)+NH4 | TG | (6:0_12:0_18:1) | (6:0) | (12:0) | (18:1) | 2.204465775 | 0.020674 | 5.378025 |
| GS | POS3597 | TG(16:0_8:0_18:1)+NH4 | TG(42:1)+NH4 | TG | (16:0_8:0_18:1) | (16:0) | (8:0) | (18:1) | 2.542692643 | 0.002342 | 3.376033 |
| GS | POS3351 | TG(16:0_6:0_18:1)+NH4 | TG(40:1)+NH4 | TG | (16:0_6:0_18:1) | (16:0) | (6:0) | (18:1) | 4.292457437 | 0.000264 | 10.14785 |
| GS | POS4376 | TG(16:0_16:0_18:1)+NH4 | TG(50:1)+NH4 | TG | (16:0_16:0_18:1) | (16:0) | (16:0) | (18:1) | 1.840078319 | 0.026722 | 7.380755 |
| GS | POS4177 | TG(16:0_14:0_18:1)+NH4 | TG(48:1)+NH4 | TG | (16:0_14:0_18:1) | (16:0) | (14:0) | (18:1) | 2.273645944 | 0.006085 | 4.095671 |
| GS | POS3790 | TG(16:0_10:0_18:1)+NH4 | TG(44:1)+NH4 | TG | (16:0_10:0_18:1) | (16:0) | (10:0) | (18:1) | 2.242268004 | 0.007968 | 4.836063 |
| GS | POS3542 | TG(18:0_6:0_18:0)+NH4 | TG(42:0)+NH4 | TG | (18:0_6:0_18:0) | (18:0) | (6:0) | (18:0) | 1.839154018 | 0.045852 | 6.629941 |
| GS | POS3981 | TG(16:0_12:0_18:1)+NH4 | TG(46:1)+NH4 | TG | (16:0_12:0_18:1) | (16:0) | (12:0) | (18:1) | 1.82738651 | 0.037343 | 6.203106 |
| GS | POS3301 | TG(4:0_18:0_18:0)+NH4 | TG(40:0)+NH4 | TG | (4:0_18:0_18:0) | (4:0) | (18:0) | (18:0) | 1.855542717 | 0.129448 | 5.602037 |
| GS | POS2528 | TG(10:0_10:0_12:0)+NH4 | TG(32:0)+NH4 | TG | (10:0_10:0_12:0) | (10:0) | (10:0) | (12:0) | 2.083005178 | 0.021925 | 5.30981 |
| GS | POS5494 | ZyE(33:6)+NH4 | ZyE(33:6)+NH4 | ZyE | (33:6) | (33:6) |  |  | 0.727341751 | 0.612189 | 8.714768 |
| GS | POS3821 | TG(16:1_10:0_18:1)+NH4 | TG(44:2)+NH4 | TG | (16:1_10:0_18:1) | (16:1) | (10:0) | (18:1) | 2.361259822 | 0.012281 | 3.682671 |
| GS | POS3390 | TG(4:0_18:1_18:1)+NH4 | TG(40:2)+NH4 | TG | (4:0_18:1_18:1) | (4:0) | (18:1) | (18:1) | 3.205109061 | 0.000265 | 4.086534 |
| GS | POS5482 | ZyE(23:6)+NH4 | ZyE(23:6)+NH4 | ZyE | (23:6) | (23:6) |  |  | 1.637327029 | 0.294017 | 7.487906 |
| GS | POS4663 | TG(16:0_18:1_18:1)+NH4 | TG(52:2)+NH4 | TG | (16:0_18:1_18:1) | (16:0) | (18:1) | (18:1) | 3.021477302 | 0.000662 | 1.713745 |
| GS | POS3057 | TG(16:0_6:0_16:0)+NH4 | TG(38:0)+NH4 | TG | (16:0_6:0_16:0) | (16:0) | (6:0) | (16:0) | 3.645751259 | 0.012446 | 4.664742 |

Note:Y= Yak milk; GS = German Simmental milk
